# Mismatch repair protein deficiency and its implications on distant metastasis in colorectal cancer: A comprehensive analysis

**DOI:** 10.1002/cam4.6994

**Published:** 2024-03-28

**Authors:** Chuanwen Fan, Chao Fang, Wei Wang, Zhaoying Lv, Xueli Zhang, Feiwu Long, Zongze Jiang, Yuan Li, Hong Zhang, Zong‐Guang Zhou, Cun Wang, Xiao‐Feng Sun

**Affiliations:** ^1^ Institute of Digestive Surgery, Department of Gastrointestinal Surgery, West China Hospital Sichuan University Chengdu China; ^2^ Department of Gastrointestinal, Bariatric and Metabolic Surgery, Research Center for Nutrition, Metabolism & Food Safety West China‐PUMC C.C. Chen Institute of Health, West China School of Public Health and West China Fourth Hospital, Sichuan University Chengdu China; ^3^ Department of Oncology and Department of Biomedical and Clinical Sciences Linköping University Linköping Sweden; ^4^ Colorectal Cancer Center, Department of General Surgery, West China Hospital Sichuan University Chengdu China; ^5^ Department of Medical Sciences Örebro University Örebro Sweden

**Keywords:** colorectal cancer (CRC), IRF1, meta‐analysis, metastasis, mismatch repair (MMR), weighted gene co‐expression network analysis (WGCNA)

## Abstract

**Background:**

While previous studies have indicated variability in distant metastatic potential among different mismatch repair (MMR) states in colorectal cancer (CRC), their findings remain inconclusive, especially considering potential differences across various ethnic backgrounds. Furthermore, the gene regulatory networks and the underlying mechanisms responsible for these variances in metastatic potential across MMR states have yet to be elucidated.

**Methods:**

We collected 2058 consecutive primary CRC samples from the South West of China and assessed the expression of MMR proteins (MLH1, MSH2, MSH6, and PMS2) using immunohistochemistry. To explore the inconsistencies between different MMR statuses and recurrence, we performed a meta‐analysis. To delve deeper, we employed Weighted Gene Co‐expression Network Analysis (WGCNA), ClueGo, and iRegulon, pinpointing gene expression networks and key regulatory molecules linked to metastasis and recurrence in CRC. Lastly, both univariate and multivariate Cox regression analyses were applied to determine the impact of core regulatory molecules on metastasis.

**Results:**

Of the samples, 8.2% displayed deficient MMR (dMMR), with losses of MLH1 and PSM2 observed in 40.8% and 63.9%, respectively. A unique 24.3% isolated loss of PMS2 without concurrent metastasis was identified, a result that diverges from established literature. Additionally, our meta‐analysis further solidifies the reduced recurrence likelihood in dMMR CRC samples compared to proficient MMR (pMMR). Two gene expression networks tied to distant metastasis and recurrence were identified, with a majority of metastasis‐related genes located on chromosomes 8 and 18. An IRF1 positive feedback loop was discerned in the metastasis‐related network, and IRF1 was identified as a predictive marker for both recurrence‐free and distant metastasis‐free survival across multiple datasets.

**Conclusion:**

Geographical and ethnic factors might influence peculiarities in MMR protein loss. Our findings also highlight new gene expression networks and crucial regulatory molecules in CRC metastasis, enhancing our comprehension of the mechanisms driving distant metastasis.

## 1 INTRODUCTION

Colorectal cancer (CRC) is the third most common malignancy worldwide.^1^ According to the latest cancer statistics in China, nearly one‐fourth of new CRC diagnoses occur in China.^2^

Recently, defective mismatch repair (dMMR) CRC has been recognized for its potential to predict prognosis and guide therapeutic decisions.^3^ While dMMR CRC typically exhibits a low prevalence of distant metastasis and generally has a favorable prognosis, its characteristics remain a topic of debate.^4^, ^5^ Although the majority of CRCs arise via a chromosomal instability pathway, around 10%–15% follow a microsatellite instability (MSI) pathway.^6^ Currently, both the US National Comprehensive Cancer Network Clinical Practice Guidelines in Oncology for Genetic/Familial High‐Risk Assessment (Colorectal) and the UK's National Institute for Health and Care Excellence (NICE) recommend universal screening of dMMR in all CRC cases.^7^ However, the implementation of this recommendation in the Chinese population, especially in a large unselected CRC series, remains under‐explored.

The relationship between DNA mismatch repair (MMR) status and metastatic potential and survival in CRC is intricate.^5^ Distant metastatic spread unquestionably worsens prognosis: approximately 50% of CRC patients will develop distant metastases, accounting for 90% of CRC‐related deaths.^5^ The 5‐year survival rates drop sharply from 90% in early‐stage patients to about 10% for those with distant metastases.^8^ Additionally, distant metastases frequently hinder effective treatment, becoming a primary reason for therapeutic failure—even when various cytotoxic and targeted therapies are administered.^9^ Consequently, distant metastasis emergence becomes a critical turning point in the disease progression. Notably, distant metastatic spread occurs in only 3.5% of dMMR CRC cases.^10^ The mechanisms behind this low propensity for distant metastasis in dMMR CRC remain poorly understood, prompting our interest in uncovering the underlying reasons.

Over recent years, numerous studies have explored the regulatory signaling pathways and the progression from normal mucosa to malignancy. High‐throughput sequencing, gene microarray techniques, and bioinformatics algorithms have offered significant insights. While various studies on dMMR have employed transcriptome analysis to provide insight into CRC, they often focus on different molecular subtypes and survival characteristics without considering the dMMR/pMMR status, leading to non‐informative conclusions.^11^, ^12^ Analysis through networks of co‐expressed genes having similar biological functions has shed light on distant metastasis‐associated biological pathways in CRC. Particularly, weighted correlation network analysis (WGCNA) has proven effective in pinpointing gene signatures closely tied to tumor clinical traits.^13^

In this study, we conducted a universal screening for dMMR patients within a large, consecutive cohort of newly diagnosed CRC patients from southwestern China. We used immunohistochemical (IHC) staining to examine MMR proteins and better understand the characteristics of dMMR CRC. Additionally, we performed a meta‐analysis to analyze the recurrence potential of dMMR. To elucidate the signaling network of dMMR that inhibits distant metastasis, we integrated several dMMR mRNA expression datasets. Using WGCNA, we aimed to identify key metastatic driver signaling networks and transcription factors in CRC.

## 2 MATERIALS AND METHODS

### 2.1 Clinical specimens

A total of 2115 consecutive primary CRC patients were initially enrolled in the present study, and all patients underwent curative surgical resection between November 2014 and April 2016 at West China Hospital of Sichuan University (Chengdu, China). Among the patients, 57 patients with a clinical diagnosis of polyposis or a history of familial adenomatous polyposis or inflammatory bowel disease were excluded. Finally, a total of 2058 eligible patients were analyzed in the present study. The clinicopathological characteristics, including sex, age, tumor location, differentiation, vascular invasion, perineural invasion, and synchronous adenoma, were obtained from surgical and pathological records, and TNM staging was performed according to the American Joint Committee on Cancer (AJCC), 2017. The mean patient age was 61 years (range, 17–104 years); 60.4% of the patients were male. A total of 19.0% were located in the proximal colon, while 81.0% were located in the distal colon and rectum. 9.0% were synchronous adenomas. A total of 11.1% had poorly differentiated/mucinous tumors, and 1.3% were stage IV. All patients provided written informed consent to participate in the study.

### 2.2 Identification of eligible studies for meta‐analysis

Our literature search for meta‐analysis aimed to identify relevant articles published in English up to December 2019. We conducted this search using the PubMed (dating back to 1949), EMBASE (from 1974), and Cochrane (from 1996) databases. The search strategy incorporated medical subject headings (MeSH) and keywords in the title and abstract, focusing on terms such as “DNA MMR”, “base pair mismatch”, “MLH1”, “MSH2”, “MSH6”, “PMS2”, “colorectal neoplasms”, and “disease‐free survival (DFS)”. Additionally, we reviewed the references of pertinent articles to ensure comprehensiveness.

Eligibility criteria for the studies included in our analysis were stringent. We considered studies that evaluated DFS, recurrence‐free survival (RFS), or distant recurrence‐free survival (DRFS) in stage I–IV CRC cases. These cases had to be stratified based on MMR status, specifically delineating between deficient MMR (dMMR) and proficient MMR (pMMR). A key requirement was that the MMR status should have been ascertained through genotyping or immunohistochemistry (IHC) testing. We excluded studies that did not detail their methodology for determining MMR status. Furthermore, studies were also excluded if the dMMR patient group consisted of fewer than 10 individuals.

To ensure the uniqueness of the data, we meticulously reviewed the names of all authors, medical centers, and the inclusion time of subjects for each publication. In instances of data duplication, particularly when reports from the same author or research team were encountered, we opted for the most recent data. Our review was limited to studies published in peer‐reviewed journals. We excluded unpublished data, review articles, editorials, letters, notes, case reports, and conference abstracts unrelated to our research focus. Additionally, we did not seek unpublished data or contact authors directly.

### 2.3 Identification of eligible gene expression data of CRC with dMMR

To maximally identify the gene expression data of CRC with dMMR, we utilized the microarray databases Gene Expression Omnibus (GEO, www.ncbi.nlm.nih.gov/geo/) and ArrayExpress (www.ebi.ac.uk/arrayexpress) as our primary data sources. Our search strategy involved a combination of keywords “CRC” or “colon cancer” combined with any of “MSI”, “Replication error”, “MMR”, “dMMR”, “loss of heterozygosity” combined with any “stage”, “recurrence”, or “metastasis”. The inclusion criteria for selecting datasets were based on the presence of MSI/MMR status, cancer stage or metastasis information in the mRNA microarray datasets. We excluded microRNA microarrays, single‐nucleotide polymorphism microarrays or studies that lacked detailed stage or MSI/MMR data.

After identifying eligible studies, we obtained the raw gene expression profile and clinical data from GEO and ArrayExpress databases. These raw data were using a robust multiarray averaging method^14^ and then quantile normalized using the “affy” and “affycoretools” packages of R software (version 3.6.0, R Foundation for Statistical Computing Vienna, Austria). We identified eligible datasets: GSE39582 and GSE39084, both derived from the Affymetrix Human Genome U133 Plus 2.0 Array (GPL570), and GSE41258, which was derived from the Human Genome U133A Array (GPL96). The GSE41258 serial matrix data were directly acquired from the GEO database and underwent log2 normalization. Additionally, we incorporated RNA sequencing datasets from TCGA level 3, which were downloaded from the UCSC Cancer browser (https://xenabrowser.net/hub/). The relevant information on dMMR CRC sample cohorts for gene expression can be found in Data S1.

Our study utilized datasets derived from various platforms and created by different groups. This diversity often introduces batch effects, which are non‐biological variations caused by technical differences in experimental conditions, platforms, or processing methods. To mitigate these potential batch effects and enhance comparability, we applied the ComBat algorithm which is widely recognized as the most effective method for counteracting batch effects.^15^

### 2.4 IHC and staining evaluation

To characterize the MMR system, IHC was performed using 5‐μm‐thick paraffin tissue sections. Sections were deparaffinized in xylene and rehydrated with a series of gradient ethanol to water. The sections were heated to boiling point in citrate buffer (pH 6.0) for 30 min to unmask antigen, followed by washing in phosphate‐buffered saline (PBS). Endogenous peroxidase activity was blocked with 3% H_2_O_2_ followed by washing three times in PBS. The sections were incubated with protein block (Dako, Carpinteria, CA) for 10 min and then incubated for 2 h with antibodies against MLH1 protein (Dako, Carpinteria, CA), MSH2 protein (Dako, Carpinteria, CA), MSH6 protein (Dako, Carpinteria, CA), and PMS2 (Dako, Carpinteria, CA). The sections were washed in PBS and then incubated with goat anti‐mouse secondary antibody (Dako, Carpinteria, CA) at room temperature for 25 min. Next, the sections were subjected to 3,3′‐diaminobenzidine tetrahydrochloride for 8 min and then counterstained with hematoxylin. Negative and positive controls were added in each staining run. All slides were scored by two independent investigators.

### 2.5 MMR status determination

MSI status was obtained from the GEO database (https://www.ncbi.nlm.nih.gov/geo/) or TCGA datasets. MSI‐H tumors or tumors without any MMR protein expression were further classified as dMMR, and both MSI‐L and MSS tumors or tumors presenting all four MMR proteins, MLH1, MSH2, MSH6 and PSM2, were classified as pMMR tumors.

### 2.6 Construction of weighted gene co‐expression network

The R package WGNCA was used for co‐expression network construction.^13^ All expressed genes in the GSE41258 dataset were separately included in these analyses. First, outlier samples were removed from the subsequent analyses (Figure S3A and Data S1). A power of 6 was selected, in accordance with networks' connectivity distributions approximating the power law, indicating that the network possesses scale‐free topology. The topological overlap distance calculated from the adjacency matrix was then clustered with average linkage hierarchical clustering. To obtain moderately large and distinct modules, we set the minimum module size to 40 genes and the minimum height for merging modules at 0.20. Second, we determined correlations among gene expression modules and clinical traits, the latter of which included sex, age, TNM stage, survival and MMR status. In addition, the association of gene significance (GS) with module membership (MM) was also assessed to identify the highly related modules that were to the phenotype.

To assess the stability of each module identified above, module preservation statistics were computed using the module preservation function implemented in WGCNA, according to a previous study.^16^ The preservation analyses were performed in two platform datasets, GSE41258 and GSE39582.

### 2.7 ClueGO‐CluePedia functional analyses

To investigate the biological role of the genes of the module identified in the present study, we used a Cytoscape plugin app, ClueGO, and CluePedia which presents enriched pathways within a network, interconnected based on kappa score.^17^ In the present analyses, GO evidence was limited in all experimental models. Terms found in the 3–8 GO interval, with at least 3 genes from the initial list representing a minimum of 3%, were selected. A *p*‐value of <0.01 or <0.05 and a kappa coefficient of 0.4 were considered threshold values.

### 2.8 Hub genes identification and validation

Hub genes were a series of genes with the highest degree of connectivity in a gene module and determined the characteristics of a module. Hub genes were defined by module connectivity, measured by the absolute value of Pearson's correlation (cor. MM >0.8) and clinical trait relationship and measured by the absolute value of Pearson's correlation (cor. GS >0.2). In the present study, we identified hub genes in the module that significantly correlated with distant metastasis. GSE39582 and TCGA datasets were analyzed to validate the expression of hub genes.

### 2.9 Enrichment analyses for transcription factors

Transcription factors for dark green and gray 60 modules were identified by the iRegulon V.1.3 plugin in Cytoscape with a minimum normalized enrichment score (NES) of 3.0 using data from 1120 ChIP‐seq tracks in the Encode database.^18^ For each transcription factor, we show the number of genes regulated by that transcription factor identified within the dark green and gray 60 modules (Data S2). The regulatory network of modules and transcription factors was plotted in Cytoscape to identify common regulators shared between modules.

### 2.10 Statistical analyses

A cross‐table analysis employing Pearson's *χ* ^2^‐test or Fisher's exact test, as appropriate, was used to determine associations between dMMR status and clinicopathological characteristics in IHC data. Two‐tailed Student's *t*‐test was performed to compare two groups of numerical values in gene profile data. *p* < 0.05 was considered statistically significant for all analyses, and all calculations were carried out using SPSS software (version 22.0; SPSS, Chicago, IL).

For the meta‐analysis, we analyzed the data using the “Meta” package in R. Heterogeneity was assessed with I‐square (*I* ^2^) and chi‐square (*χ*2) tests. If *I* ^2^ >50% or *p* ≤ 0.05, a random effects model was used; otherwise, a fixed effects model was applied. We explored sources of heterogeneity through subgroup analysis, meta‐regression, and Galbraith plot. To check for publication bias, we used funnel plots, Begg and Egger's test. Sensitivity analysis determined result stability.

To evaluate the impact of core transcription factors on RFS and DMFS in CRC patients, univariable and multivariable analyses were performed by Cox regression using the “survival” package in R 3.6.0. Only GSE39084 datasets were analyzed for DMFS because DMFS data were available.

## 3 RESULTS

### 3.1 Association of MMR status with specific clinicopathological characteristics

**FIGURE 1:**
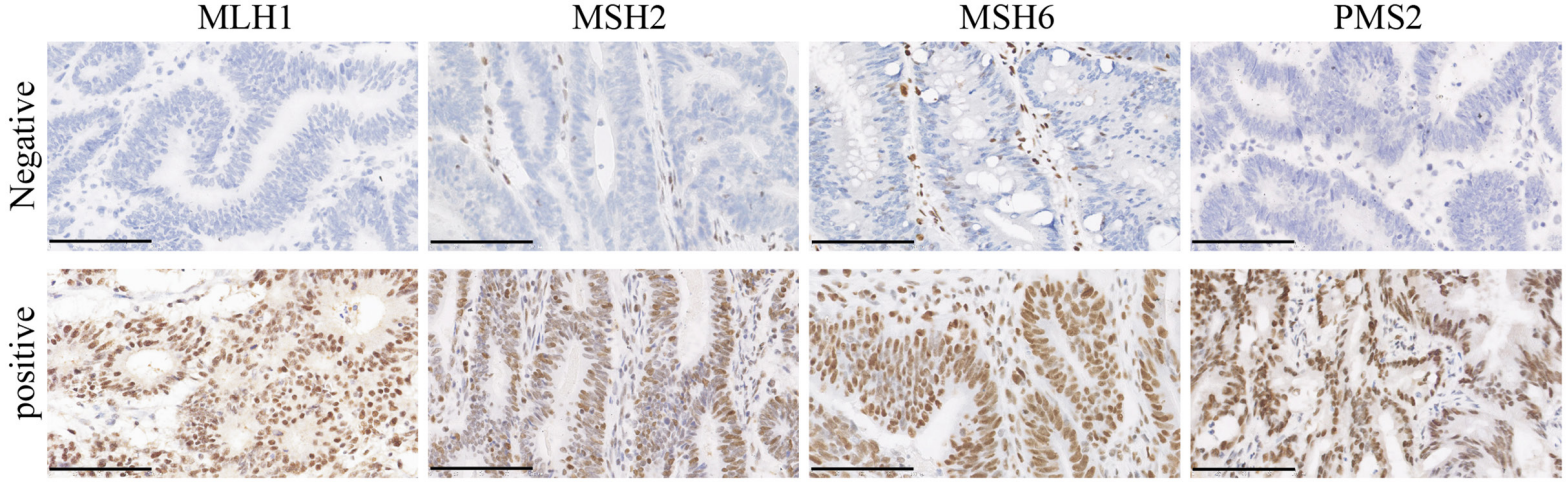
The representative image of MMR protein expression. Representative images of negative and positive expression of MLH1, MSH2, MSH6, and PSM2 (scale bar: 100 μm).

The expression of MMR proteins MLH1, MSH2, MSH6, and PSM2 was examined across all 2058 CRC samples (Figure 1). Out of these, 169 patients exhibited a lack of expression for at least one of the proteins (Table 1). As detailed in Table 1, patients with dMMR were significantly younger than those with pMMR, having a mean age of 55.8 ± 15.5 vs. 61.8 ± 12.4 (*p* < 0.001). dMMR was more prevalent in cancers originating from the proximal colon than those from the distal colon and rectum (53.8% vs. 46.2%, *p* < 0.001). Moreover, dMMR showed a stronger association with poorly differentiated or mucinous histology (23.1% vs. 10.1%, *p* < 0.001) and was less frequently associated with distant metastasis (0.0% vs. 1.4%, *p* = 0.043) than pMMR. However, no significant differences between dMMR and pMMR were observed in terms of vascular invasion, perineural invasion, or the presence of synchronous adenoma (Table 1).

**TABLE 1:** Mismatch repair proteins by immunohistochemistry in relation to clinicopathological characteristics in colorectal cancer patients.

Parameters	*N* (2058)	MMR protein status^a^		*χ* ^2^	*p*
		pMMR (*n*/%)	dMMR (*n*/%)		
Gender					
Male	1244 (60.4)	1151 (60.9)	93 (55.0)	2.26	0.133^c^
Female	814 (39.6)	738 (39.1)	76 (45.0)		
Age (mean ± SD)	61.3 ± 12.8	61.8 ± 12.4	55.8 ± 15.5		< 0.001^b^
≤68 years	1412 (20.7)	1283 (67.9)	129 (76.3)	5.10	0.024^c^
>68 years	646 (79.3)	606 (31.1)	40 (23.7)		
Primary tumor location					
Proximal	391 (19.0)	300 (15.9)	91 (53.8)	145.28	< 0.001^c^
Distal/rectal	1667 (81.0)	1589 (84.1)	78 (46.2)		
TNM stage				38.13	< 0.001^d^
I/II	998 (18.4)	998 (52.9)	130 (76.0)		
III	904 (43.9)	865 (45.8)	39 (23.1)		< 0.001^c^
IV	26 (1.3)	26 (1.4)	0 (0.0)		0.043^d^
Differentiation					
Well/moderately	1829 (88.9)	1699 (89.9)	130 (76.9)	25.59	< 0.001^c^
Poorly/mucinous	229 (11.1)	190 (10.1)	39 (23.1)		
Vascular invasion					
Yes	256 (12.4)	233 (12.3)	23 (13.6)	0.23	0.630^c^
No	1802 (87.6)	1656 (87.7)	146 (86.4)		
Perineural invasion					
Yes	266 (12.9)	251 (13.3)	15 (8.9)	2.68	0.101^c^
No	1792 (87.1)	1638 (86.7)	154 (91.1)		
Synchronous adenoma					
Yes	186 (9.0)	175 (9.3)	11 (6.5)	1.43	0.231^c^
No	1872 (91)	1714 (90.7)	158 (93.5)		

^a^ MMR proteins included in MLH1, MSH2, MSH6, and PSM2 and the absence of any MMR protein expression were classified as dMMR; otherwise, they were classified as pMMR.

^b^ Student *t*‐test.

^c^ *p* values were calculated using Pearson *χ* ^2^ test.

^d^ *p* values were calculated using Fisher's exact test because the sample of dMMR CRC with stage IV disease had an expected count of less than 5.

Specifically, MLH1 was absent in 69 patients (40.8%), MSH2 in 48 patients (28.4%), MSH6 in 41 patients (24.3%), and PMS2 in 108 patients (63.9%). Additionally, 10 patients (5.9%) exhibited an isolated loss of MSH6, while 41 patients (24.3%) had an isolated loss of PMS2. The combined loss of MLH1 and PSM2 was observed in 66 patients (39.1%), MSH2 and MSH6 in 28 patients (16.6%), MLH1 and MSH6 in two patients (1.2%), MSH6 and PSM2 in three patients (1.2%), and a combination of MLH1, MSH6, and PSM2 was seen in two patients (1.2%) (Table 2).

**TABLE 2:** Expression of mismatch repair proteins by immunohistochemistry in 169 colorectal cancer patients with deficient mismatch repair.

MMR proteins	Present cases (*n*/%)	Absent cases (*n*/%)
MLH1	100 (59.2)	69 (40.8)
MSH2	121 (71.6)	48 (28.4)
MSH6	128 (75.7)	41 (24.3)
PSM2	61 (36.1)	108 (63.9)
Isolated MSH6	159 (94.1)	10 (5.9)
Isolated PSM2	128 (75.7)	41 (24.3)
MLH1/PSM2	103 (60.9)	66 (39.1)
MSH2/MSH6	141 (83.4)	28 (16.6)
MLH1/MSH6	167 (98.8)	2 (1.2)
MSH6/PSM2	166 (98.2)	3 (1.8)
MLH1/MSH6/PSM2	167 (98.8)	2 (1.2)

### 3.2 dMMR is associated with less risk of recurrence in CRC

**FIGURE 2:**
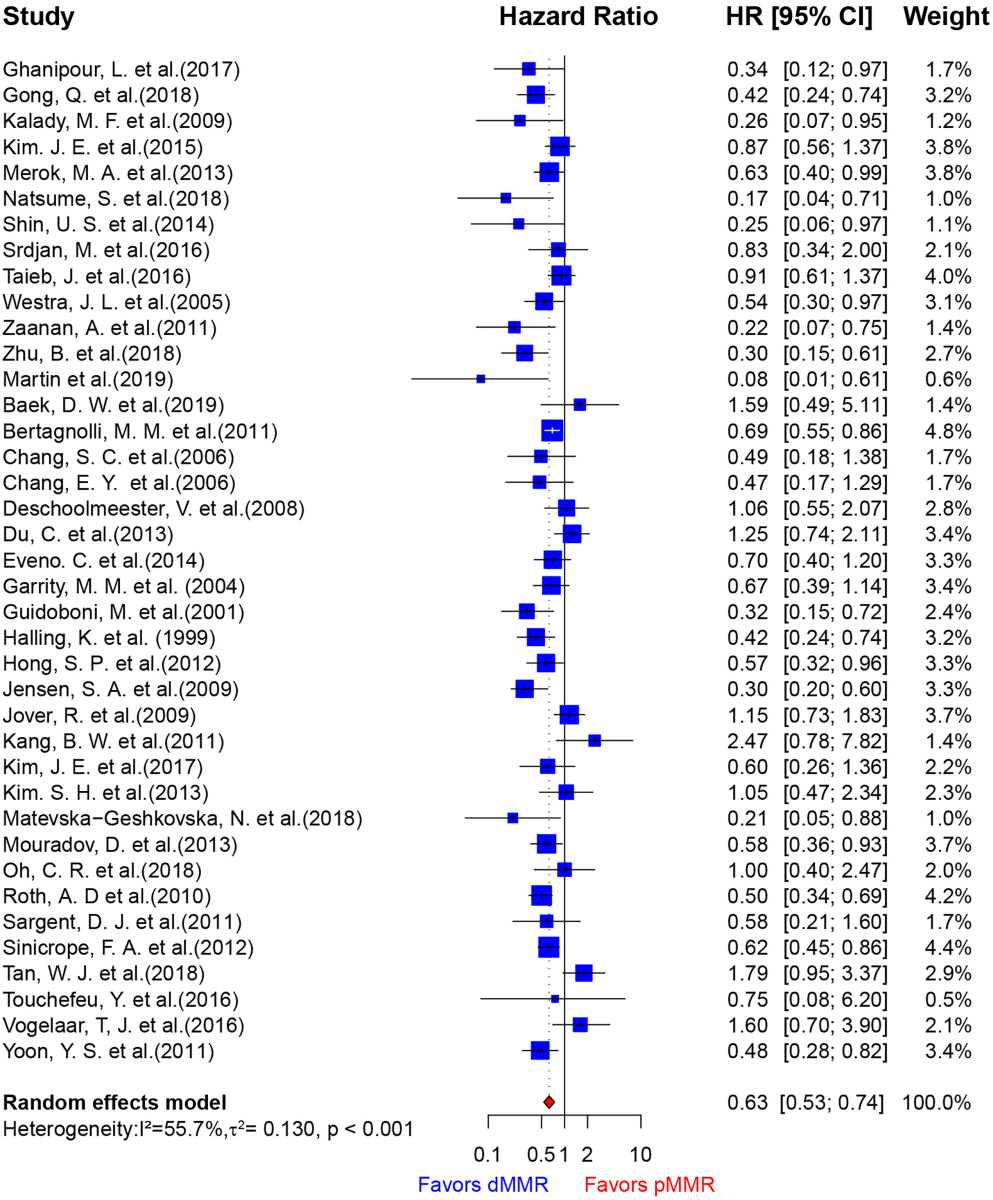
Forest plots of HRs for OS from all eligible studies of CRC associated with mismatch repair deficiency.

To comprehensively evaluate the influence of different MMR statuses on the risk of recurrence of CRC, we conducted a meta‐analysis. In this analysis, 39 studies were included eligible, encompassing 22,189 patients, of which 2949 had dMMR (Figure S1A and Table S1). We meticulously assessed the possibility of publication bias employing several methods including Begg's test, Egger's regression, and Funnel plot analyses, and our evaluations did not reveal any indications of such bias (Figure S1B–S1D). However, we observed significant heterogeneity among the studies (*I* ^2^ = 56%, Cochran's Q *p* < 0.001). Given the presence of substantial heterogeneity, we employed a random effects model for our analysis. The result revealed that dMMR status was associated with a significantly reduced risk of recurrence (Figure 2, HR = 0.63, 95% CI: 0.53–0.74). To identify potential sources of this heterogeneity, we conducted a meta‐regression analysis and found that the stage was statistically significant (*p* < 0.001). But it only could explain about 29.08% of the between‐study variance (Table S2). Further, a Galbraith plot was created to further graphically assess the sources of heterogeneity (Figure S1E), and eight outlying studies were identified. Once these outlying studies were excluded, the heterogeneity was effectively decreased (Figure S2A, *I* ^2^ = 19.8%, Cochran's Q *p* = 0.166). However, the new pooled estimate did not change essentially (Figure S2A, Fixed effect model: HR = 0.62, 95% CI: 0.56–0.68, *p* < 0.001; Random effects model: HR = 0.610, 95% CI: 0.55–0.68, *p* < 0.001). Additionally, sensitivity analysis confirmed the robustness of our findings, as no individual study significantly altered the overall effect (Figure S2B).

### 3.3 Identification of distant metastasis and recurrence‐related gene network in CRC patients with different MMR status using WGCNA

A co‐expression network was initially constructed using all expressed genes (*n* = 12,413) from an independent dataset, GSE41258, which includes 168 samples (Figure S3A). As shown in Figure S3B, a power of 6 was selected to produce a hierarchical clustering tree (dendrogram). Subsequently, through dynamic tree cutting and merged dynamics, 26 gene modules were identified (Figure S3C, Table S3). The correlation of each module with all available clinical traits in GSE41258 was then analyzed, with a *p*‐value significance calculated for each trait‐module pairing.

To assess the stability of the indicated modules, we conducted a module preservation analysis by comparing the GSE41258 dataset on the GPL96 platform against the GSE39582 dataset on the GPL570 platform. The results revealed that the distant metastasis‐related modules (darkgreen and gray60) were stably preserved in the GSE39582 dataset, as indicated by their high Zsummary statistics (Figure S4).

**FIGURE 3:**
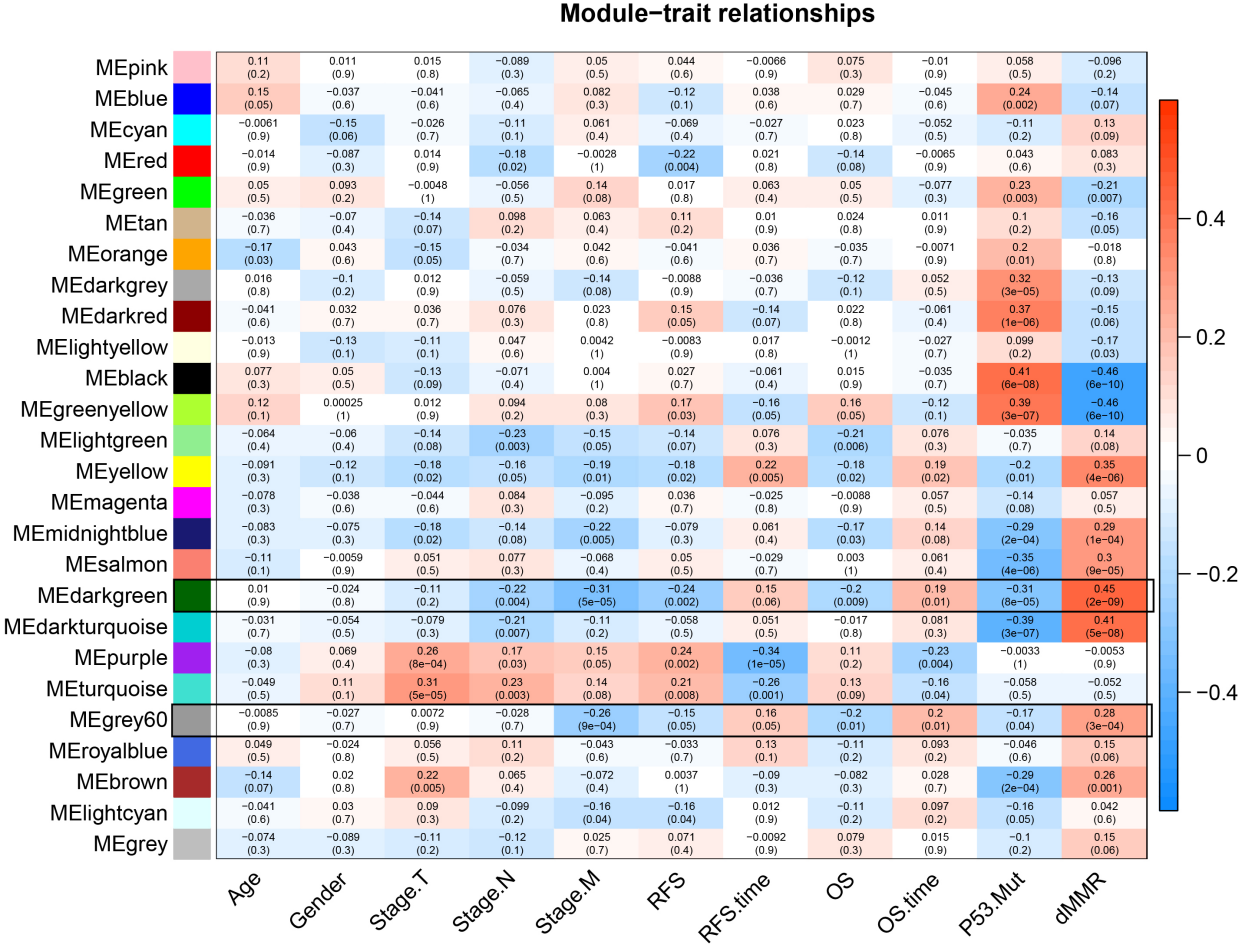
Weighted gene co‐expression network analysis heatmap. Using the costumed parameter settings and all expressed genes (*n* = 12,413), 26 gene modules were identified using weighted gene co‐expression network analyses that were correlated with clinical and molecular traits.

As shown in Figure 3 and Table S3, six modules that positively correlated with dMMR were identified, including darkgreen (cor = 0.45; *p* = 2e‐09), darkturquoise (cor = 0.41; *p* = 5e‐08), yellow (cor = 0.35; *p* = 4e‐06), salmon (cor = 0.30; *p* = 9e‐05), midnightblue (cor = 0.29; *p* = 1e‐04), and gray60 (cor = 0.28; *p* = 3e‐04) (Figure 3). The darkgreen module (*N* = 76 genes) showed strong negative correlations with stage M (cor = −0.31; *p* = 5e‐05) and p53 mutation (cor = −0.31; *p* = 8e‐05), and modest negative correlations with stage N (cor = −0.22; *p* = 0.004), RFS (cor = −0.24; *p* = 0.002), and overall survival (OS) status (cor = −0.20; P = 0.009). The darkturquoise module (*N* = 71 genes) was negatively associated with stage N (cor = −0.21; *p* = 0.007) and p53 mutation (cor = −0.39; *p* = 3e‐07). The yellow module (*N* = 1071 genes) had a modest positive association with RFS time (cor = 0.22; *p* = 0.005). The salmon module (*N* = 172 genes) showed a strong negative correlation with the p53 mutation (cor = −0.35; *p* = 4e‐06). The midnightblue module (*N* = 144 genes) had modest negative correlations with stage M (cor = −0.22; *p* = 0.005) and p53 mutation (cor = −0.29; *p* = 2e‐04). Lastly, the gray60 module (*n* = 114 genes) exhibited a negative correlation with stage M (cor = −0.26; *p* = 9e‐04). Additionally, two modules were negatively correlated with dMMR: black (cor = −0.46; *p* = 6e‐10) and greenyellow (cor = −0.46; *p* = 6e‐10) (Figure 3). The black module (N = 579 genes) demonstrated a high positive correlation with p53 mutation (cor = 0.41; *p* = 6e‐08), while the greenyellow module (N = 226 genes) also had a positive correlation with p53 mutation (cor = 0.39; *p* = 3e‐07).

### 3.4 Network of the gene modules related to metastasis

Given the above results which suggested that fewer CRCs with dMMR developed distant metastasis and recurrence compared to those with pMMR, subsequent analysis focused on the module‐trait correlations of highest significance related to both simultaneous distant metastasis and recurrence. Among the eight gene modules described in Figure 3 (six modules positively and two negatively correlated with dMMR), both the darkgreen and gray60 modules showed positive correlations with dMMR but were strongly inversely correlated with distant metastasis. As such, these two modules, darkgreen and gray60, were further explored as potential metastasis‐associated gene clusters of interest.

The darkgreen module contained 76 genes, while the gray60 module had 114 genes. Intriguingly, 92% (70/76) of the genes in the dark green module were located on chromosome 8 (47/76 genes) and chromosome 18 (23/76 genes). In contrast, the genes in the gray60 module were distributed across various chromosomes (Table S4). This distinct chromosomal distribution between the darkgreen and gray60 modules suggests these genes likely have different functional roles.

**FIGURE 4:**
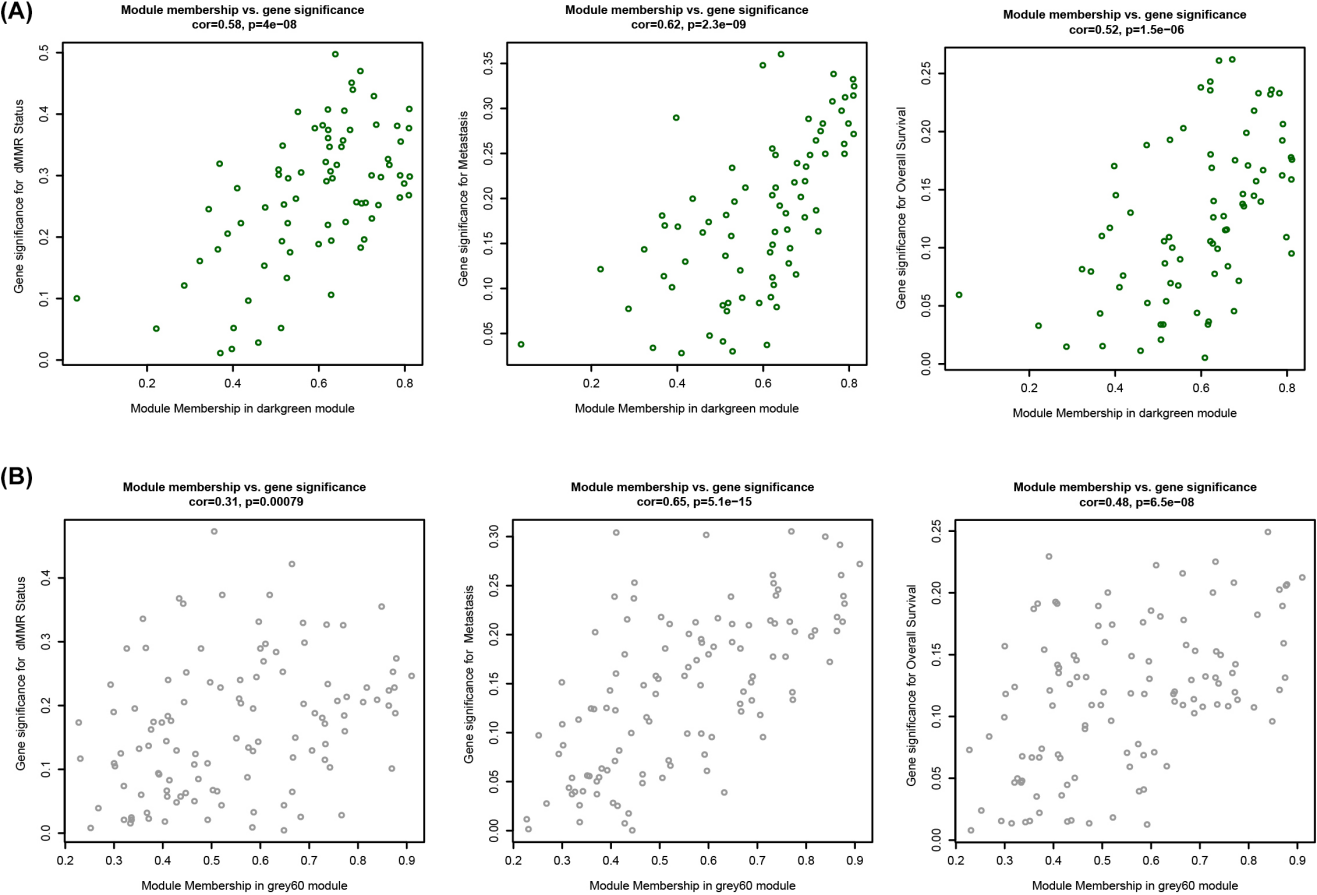
The absolute gene significance (GS) versus module membership (MM) of metastasis‐related gene modules. Weighted gene co‐expression network analysis calculation of GS to sample traits versus MM. In oversimplified terms, MM is a measure of how “tight” genes cluster within the module or, mathematically, how close gene expression is to the module eigenvalue. A gene with high MM and GS identifies hub genes that are both key components of the underlying biological process and highly correlated with the trait of interest. (A) MM was plotted against GS of the dark green module for dMMR status, metastasis, and overall survival. (B) MM was plotted against GS of the gray60 module for dMMR status, metastasis, and overall survival. cor, correlation or associations of modules with individual traits as calculated by weighted gene co‐expression network analyses software utilizing standard Pearson's correlation.

To delve deeper into the gene composition of the metastasis‐related darkgreen and gray60 modules, GS and module membership (MM) analyses—highly relevant to dMMR and metastasis—were conducted. Both the darkgreen and gray60 modules displayed significant correlations between MM and GS. Specifically, both modules had strong correlations in MM with respect to dMMR (cor = 0.58; *p* = 4.0e‐08 for darkgreen and cor = 0.31; *p* = 7.9e‐04 for gray60) and metastasis (cor = 0.62; *p* = 2.3e‐09 for darkgreen and cor = 0.65; *p* = 5.1e‐15 for gray60) (Figure 4A,B). These findings suggest that the genes within the darkgreen and gray60 modules play a pivotal role in the metastatic progression of CRC.

### 3.5 Functional enrichment of the metastasis‐related modules

**FIGURE 5:**
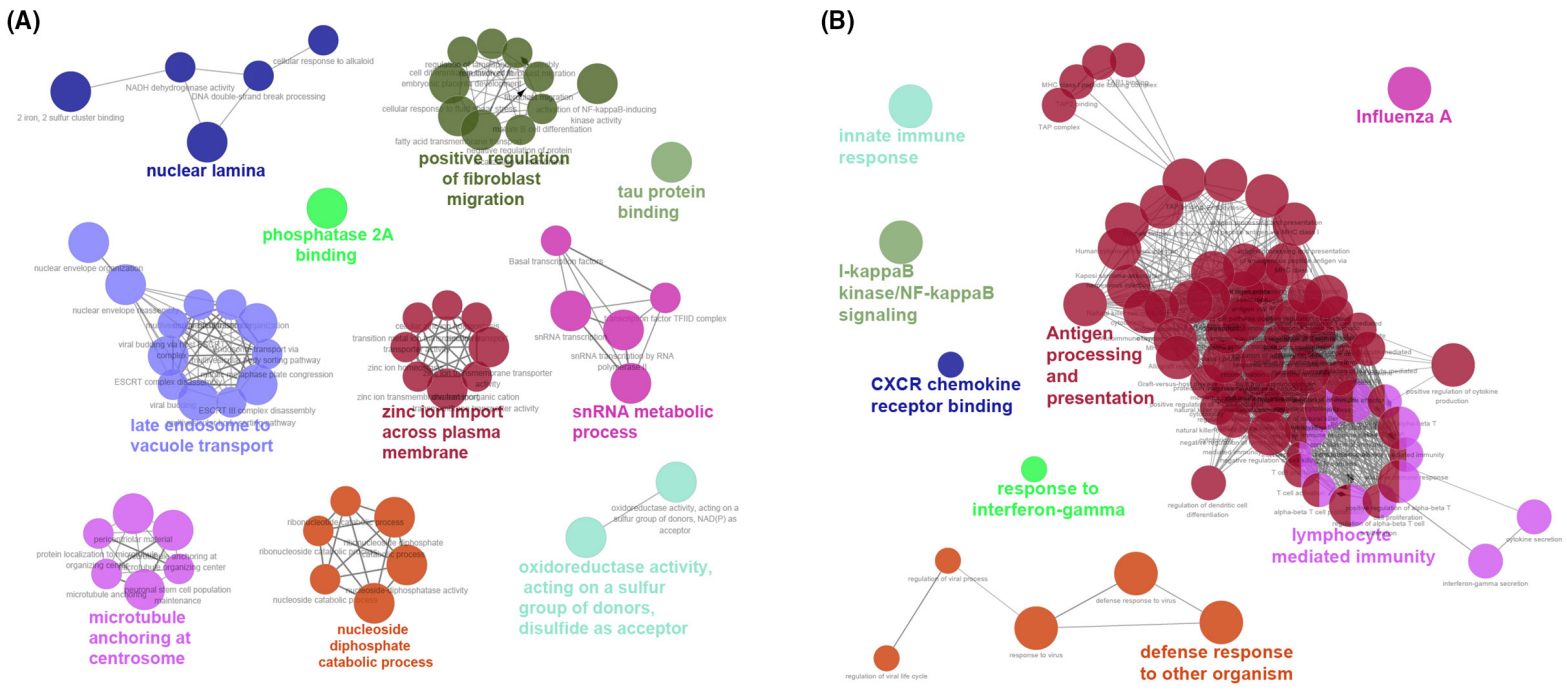
Integrative analyses of GO functions and pathways for metastasis‐related module genes. (A) Enriched functions and pathways of the metastasis‐related dark green module genes. The pathways are functionally grouped and interconnected based on the kappa score. The size of the nodes shows the term significance after Benjamini‐Hochberg correction (*p* < 0.05). (B) Enriched functions and pathways of the metastasis‐related gray60 module genes. The pathways are functionally grouped and interconnected based on the kappa score. The size of the nodes shows the term significance after Bonferroni correction (*p* < 0.01).

To delve deeper into the significance of the metastasis‐related darkgreen and gray60 modules, we conducted GO term enrichment analyses and pathway analyses using ClueGo. As depicted in Figure 5A and detailed in Table S5, the gene functions and pathways of the darkgreen module were primarily related to tumor metastasis, including positive regulation of fibroblast migration (*p* = 3.34e‐05), nuclear lamina (*p* = 8.99e‐05), oxidoreductase activity (*p* = 4.09e‐04), late endosome to vacuole transport (*p* = 4.83e‐04), microtubule anchoring at the centrosome (*p* = 5.23e‐04), protein phosphatase 2A binding (*p* = 5.99e‐04), snRNA metabolic process (*p* = 1.47e‐03), tau protein binding (*p* = 2.18e‐03), zinc ion import across the plasma membrane (P = 1.60e‐02), and nucleoside diphosphate catabolic process (*p* = 2.37e‐02).

Conversely, as presented in Figure 5B and Table S6, the genes enriched in the gray60 module were predominantly linked to immunological activities, including antigen processing and presentation (*p* = 1.57e‐13), lymphocyte‐mediated immunity (*p* = 3.60e‐09), innate immune response (*p* = 1.08e‐08), defense response to other organisms (*p* = 9.70e‐08), influenza A (*p* = 1.09e‐05), I‐kappaB kinase/NF‐kappaB signaling (*p* = 2.49E‐05), CXCR chemokine receptor binding (*p* = 3.14e‐04), and response to interferon‐gamma (*p* = 4.52e‐04).

Combined with our previous findings, these results suggest that metastatic susceptibility in CRC may be influenced by the regulation of processes such as positive fibroblast migration, nuclear lamina dynamics, antigen processing, and lymphocyte‐mediated immune responses.

### 3.6 Identification and validation of hub genes associated with dMMR and metastasis in CRC

To explore the hub genes of metastasis‐related modules, we selected genes that had high GS and MM for both dMMR and metastasis. The results demonstrated the highest degree of connectivity in the darkgreen and gray60 modules. In the darkgreen module, the four hub genes, all located on chromosome 8, were: general transcription factor IIE subunit 2 (GTF2E2), leptin receptor overlapping transcript like 1 (LEPROTL1), potassium channel tetramerization domain containing 9 (KCTD9), and protein phosphatase 2 regulatory subunit Balpha (PPP2R2A). For the gray60 module, the 10 hub genes were: apolipoprotein L3 (APOL3), C‐X‐C motif chemokine ligand 10 (CXCL10), C‐X‐C motif chemokine ligand 11 (CXCL11), guanylate binding protein 1 (GBP1), interferon regulatory factor 1 (IRF1), proteasome subunit beta 9 (PSMB9), retinoic acid receptor responder 3 (RARRES3), signal transducer and activator of transcription 1 (STAT1), transporter 1, ATP binding cassette subfamily B member (TAP1), and ubiquitin conjugating enzyme E2 L6 (UBE2L6) (Table S7).

**FIGURE 6:**
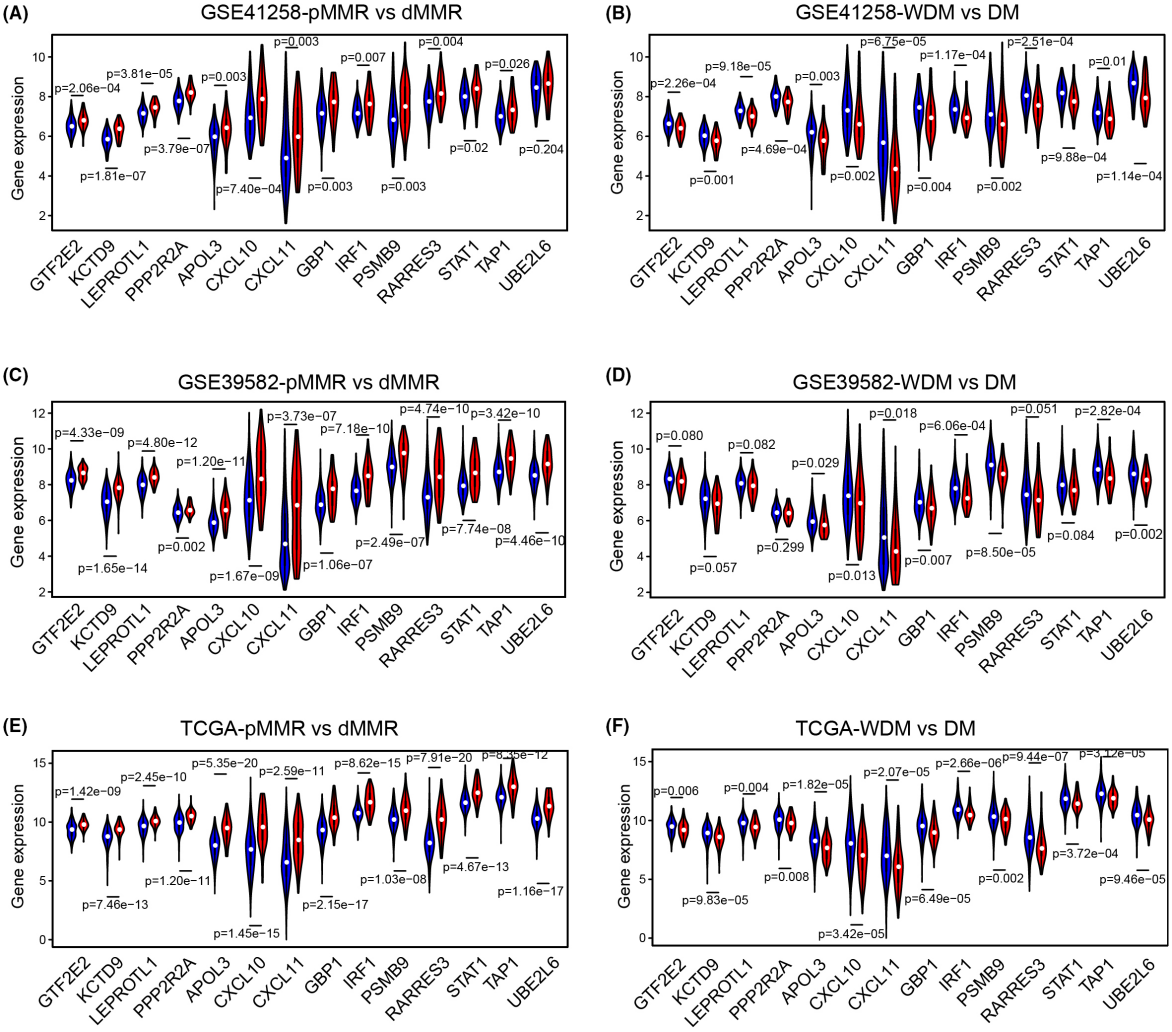
The expression of hub genes for different MMR statuses and distant metastases in different datasets. (A) The expression of each hub gene (dark green and gray 60 modules) in patients with pMMR and dMMR in GSE41258. (B) The expression of each hub gene (dark green and gray 60 modules) in patients without distant metastasis (WDM) and with distant metastasis (DM) in GSE41258. (C) The expression of each hub gene (dark green and gray 60 modules) in patients with pMMR and dMMR in the GSE39582 dataset. (D) The expression of each hub gene (dark green and gray 60 modules) in patients without distant metastasis (WDM) and with distant metastasis (DM) in the GSE39582 dataset. (E) The expression of each hub gene (dark green and gray 60 modules) in patients with pMMR and dMMR in TCGA datasets. (F) The expression of each hub gene (dark green and gray 60 modules) in patients without distant metastasis (WDM) and with distant metastasis (DM) in TCGA datasets. P‐values are the results of independent sample t‐test.

To validate the correlation between these hub genes and MMR status or distant metastasis, the expression of each hub gene was investigated separately in GSE41258 and other independent datasets, including GSE39582 and TCGA. Figure 6A showed that all hub genes were significantly upregulated in CRC patients with dMMR in the GSE41258 dataset. Conversely, all hub genes were significantly downregulated in CRC patients with distant metastasis (Figure 6B). Remarkably, similar patterns were observed across three independent datasets, despite variations in both microarray platforms (GSE41258 on the GPL96 platform, and both GSE39582 and GSE39084 on the GPL570 platform) and gene expression detection methods (employing RNA sequencing for TCGA and DNA microarray for the remaining datasets) (Figures 6C–6F). This consistent pattern underscores the robust differential expression of these hub genes.

### 3.7 Finding the central role of IRF1 in the transcriptional regulation of metastasis‐related gene modules

**FIGURE 7:**
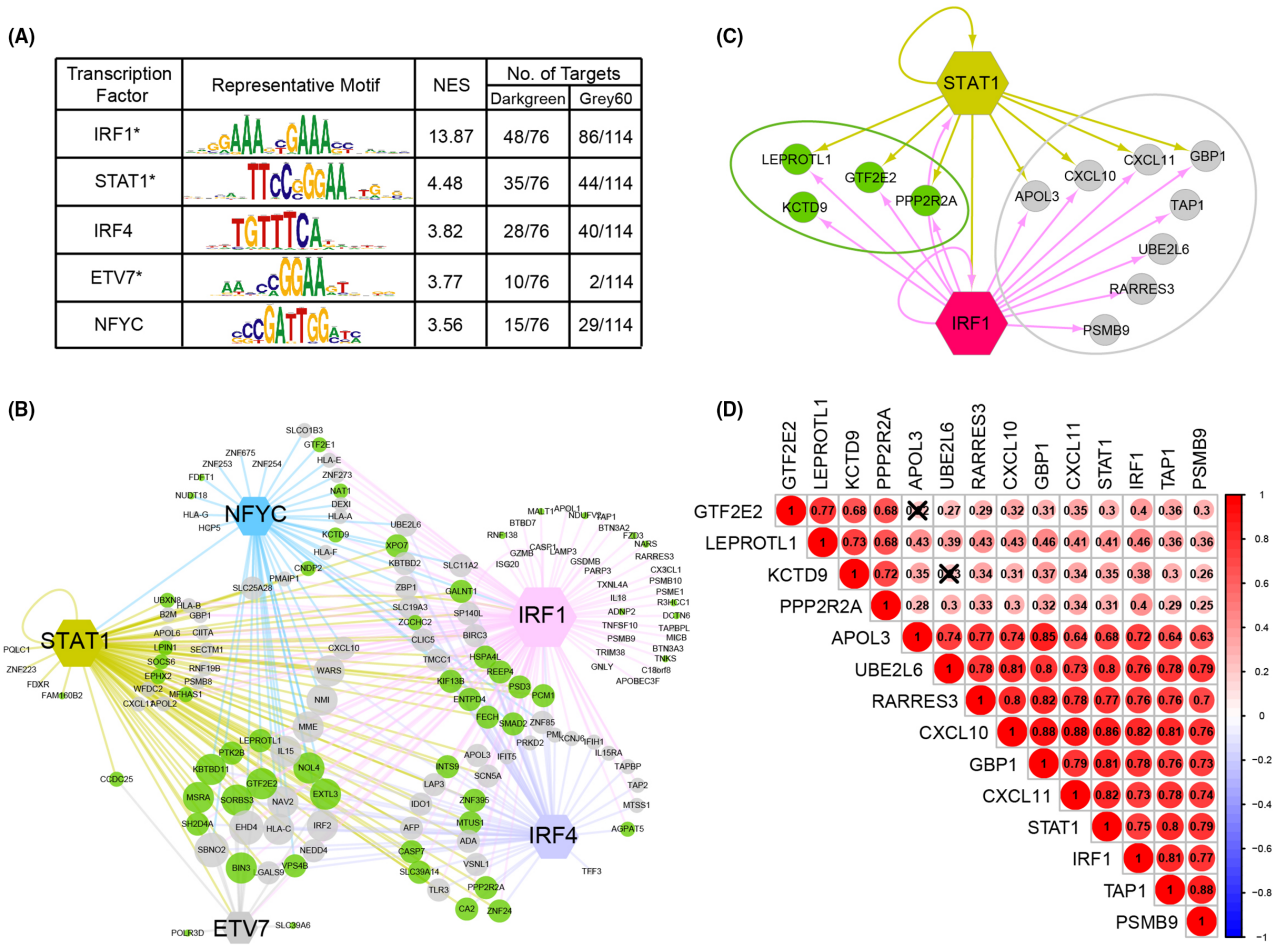
A highly interconnected network of a few transcription factors to regulate metastasis‐related gene signatures. (A) The regulatory results of transcription factors from the genes of the metastasis‐related module by iRegulon analyses (dark green and gray 60). One representative motif with the corresponding transcription factors is shown. The associated normalized enrichment score (NES) and number of predicted targets associated with each transcription factor cluster are indicated for the three independent analyses. (B) Genetic network controlling the metastasis‐related gene signature derived from iRegulon. Hexagon represents a transcription factor. Darkgreen nodes represent the genes from darkgreen module. Gray nodes represent the genes from gray60 module. Arrows colored according to the regulating transcription factor indicate predicted direct regulation. (C) Cross‐regulation among the network transcription factors and regulated to core genes of darkgreen and gray60. Darkgreen nodes represent the genes from darkgreen module. Gray nodes represent the genes from gray60 module. Arrows colored according to the regulating transcription factor indicate predicted direct interactions. (D) Correlation analyses of transcription factors and core genes of dark green and gray 60. The color bar represents the correlation coefficient, and the size of the nodes shows the significance of the correlation (*p* < 0.001); otherwise, the nodes (*p* > 0.001) are marked with a cross.

To decipher the coordinated regulation of the co‐expressed genes in the darkgreen and gray60 modules, the iRegulon framework was employed. This tool pinpoints transcription factor‐binding motifs enriched in the genomic regions of a given gene set, thereby predicting the transcription factors that potentially bind to these motifs. From this analysis, 70 motifs were found to be significantly enriched (NES > 3.0). These motifs subsequently clustered into 18 distinct groups of transcription factors based on similarity, culminating in a prediction of 77 transcription factors (Data S2). To streamline the selection of these factors, a threshold was applied, retaining only those whose highest‐ranking motif had an NES over 3.5 across all 18 motif clusters. This led to the identification of five transcription factors, including IRF1, signal transducer and activator of transcription 1 (STAT1), interferon regulatory factor 4 (IRF4), ETS variant 7 (ETV7), and nuclear transcription factor Y subunit gamma (NFYC) (Figure 7A). Collectively, these five transcription factors were forecasted to directly regulate an overwhelming majority, 79% (151/190), of the genes of the tumor metastasis‐related signature (darkgreen and gray60 modules) (Data S2).

A noteworthy observation was that three of the transcription factors (IRF1, STAT1, and EVT7) were also constituents of metastasis‐related modules. These factors have the potential to regulate 75% of the metastasis‐related genes (142/190) (Data S2). Further emphasis was placed on the enrichment scores of the transcription factors IRF1 (NES = 13.87) and STAT1 (NES = 4.48). They were anticipated to govern 71% (135/190) and 42% (79/190) of the genes present in the two metastasis‐related modules, respectively. In essence, IRF1 and STAT1 emerged as central regulatory entities, with the majority of the genes anticipated to be coregulated by these two factors (Figure 7B).

An intriguing aspect was the interaction and co‐regulatory capabilities of IRF1 and STAT1, especially concerning metastasis‐related hub genes. While IRF1 influenced all 14 of the hub genes, only 8 of these genes were also regulated by STAT1 (8/14) (Figure 7C). Correlational analysis further emphasized this relationship, revealing both IRF1 and STAT1 to be strongly positively associated with the metastasis‐related hub genes (Figure 7D). Conclusively, these results underscored the pivotal roles of IRF1 and STAT1 in the metastasis modules. Among the two, IRF1 appeared to be more influential, potentially serving as a more core transcription factor for distant metastasis.

### 3.8 Prognostic significance of IRF1 in CRC metastasis

**FIGURE 8:**
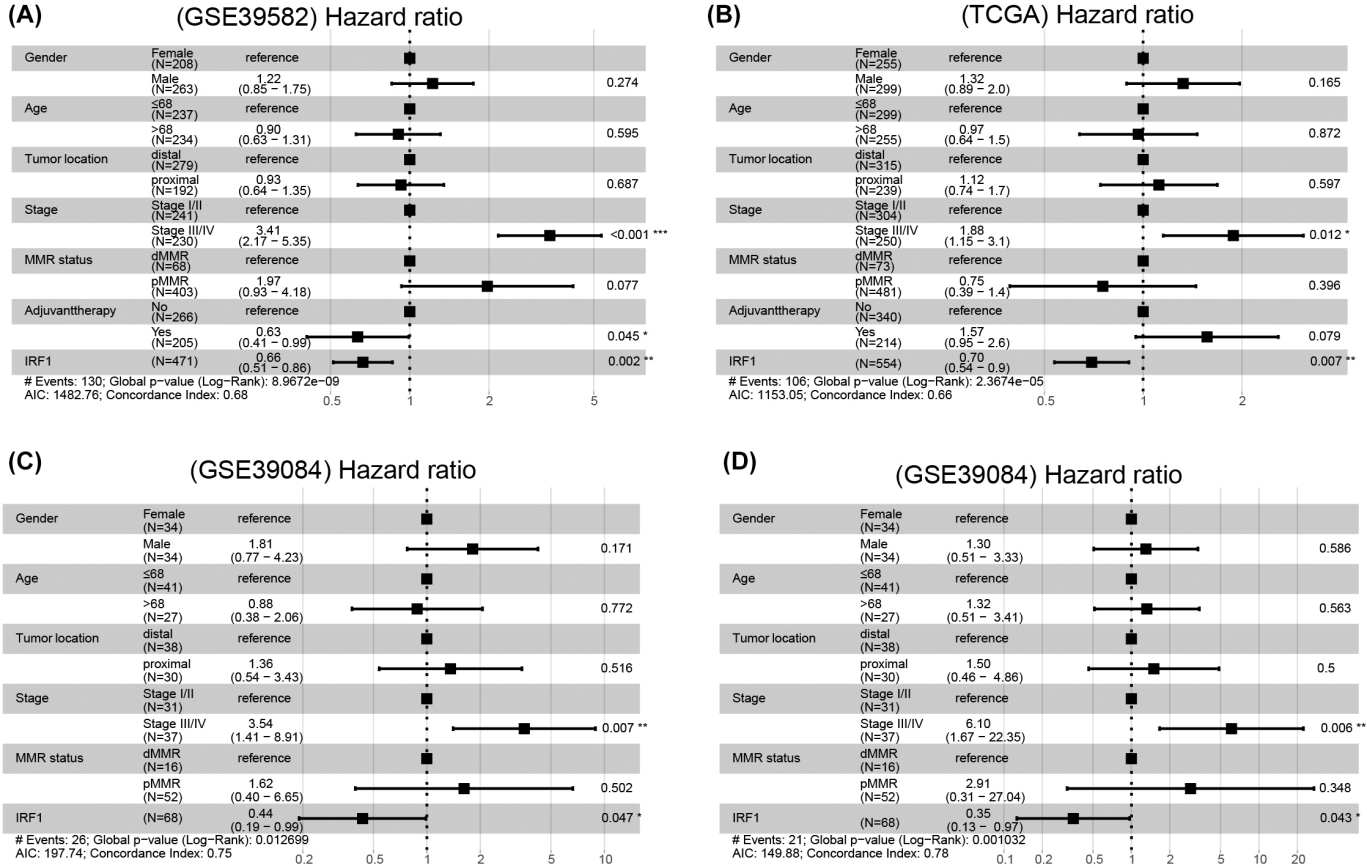
Forest plot summary of analyses of recurrence‐free survival (RFS) and distal metastasis‐free survival (DMFS). (A) Multivariable analyses for RFS of sex, age, tumor location, tumor stage, MMR status, adjuvant chemoradiotherapy and IRF1 in the GSE39582 dataset. (B) Multivariable analyses for RFS of sex, age, tumor location, tumor stage, MMR status, adjuvant chemoradiotherapy and IRF1 in the TCGA dataset. (C) Multivariable analyses of RFS by sex, age, tumor location, tumor stage, MMR status and IRF1 in the GSE39084 dataset. (D) Multivariable analyses for DRFS of sex, age, tumor location, tumor stage, MMR status and IRF1 in the GSE39084 dataset. The squares on the transverse lines represent the hazard ratio (HR), and the transverse lines represent the 95% CI.

Given the prominent expression of IRF1 in CRC patients with distal metastasis and its extensive regulatory influence on metastasis‐related module genes (Figures 6 and Figure 7), we delved deeper into the prognostic value of IRF1. Univariable and multivariable Cox regression analysis were conducted, accounting for covariates such as sex, age, tumor location, stage, adjuvant chemoradiotherapy, and MMR status. In the univariate Cox analysis, IRF1 was significantly associated with RFS, presenting an HR of 0.64 (Data S3, *p* = 3.89e‐04). This significance persisted in the GSE39582 dataset even after adjustments, with IRF1 showing an HR of 0.66 (*p* = 0.002) (Figure 8A). Further validation in the TCGA dataset corroborated the prognostic relevance of IRF1 in RFS with an HR of 0.70 (*p* = 0.005). Post‐adjustment for covariates, the impact of IRF1 on RFS remained significant (HR = 0.70, *p* = 0.007) (Figure 8B, Data S3).

Turning our focus to the GSE39084 dataset, which comprises both RFS and DMFS data, univariate Cox analysis demonstrated a significant association between IRF1 and RFS (Data S3, HR = 0.48, *p* = 0.026). This association persisted in multivariate Cox analysis with an HR of 0.44 (*p* = 0.047) (Figure 8C). Notably, IRF1 was also considerably linked with DMFS in univariate Cox analysis (HR = 0.35, *p* = 0.008), and this relationship remained significant even after adjusting for other variables, presenting an HR of 0.35 (Figure 8D, *p *= 0.043). These findings underscore IRF1 as an independent prognostic marker for DMFS, suggesting its pivotal role in the distant metastasis of CRC.

## 4 DISCUSSION

Given its potential in guiding prognosis and therapeutic decisions, including choices about adjuvant chemotherapy or immunotherapy, MMR or MSI testing is recommended for all CRC patients. However, data regarding Chinese patients with CRC are scarce. The only universal screening for dMMR was from southern China.^7^, ^19^ The screening of dMMR in populations from western China with different ethnic group distributions and lifestyles has not been well investigated, especially in a large unselected CRC series. Therefore, our results could provide great supplementary fundamental data for direct implications in clinical practice.

In our study, 169 (8.2%) patients were found to have tumors with dMMR. The incidence of dMMR CRCs was lower than that in populations from Western countries and even from southern China.^7^ These differences might be explained by the variation in the epigenetic background among populations. There is a greater difference in ethnic distribution between western China and the southern China region. The proportion of MLH1 absence was 70%–80% of all dMMR tumors in the Western population^20^ and 47.9% in the southern China population.^7^ However, it accounted for only 40.8% in our Western China cohort. Notably, 63.9% absence of PSM2 was found in our results. MLH1 and PMS2 proteins are often lost together. The higher proportion of loss of PMS2 protein than MLH1 generally means some of the patients had isolated loss of PMS2, indicating underlying germline PMS2 mutation.^3^ However, the isolated loss of PMS2 expression was thought to be an uncommon phenotype in CRCs, accounting for approximately 4% of tumors with MSI in the Western population^21^ and 7.9% in southern China.^7^ Further analysis of the clinical features and reasons behind the high proportion of isolated PMS2 loss will be valuable. Another apparent difference is that a lower proportion of proximal CRC was reported in our data, a common incidence pattern in a Chinese cohort, which is different from Western countries.^22^ In addition, no synchronous metastasis was diagnosed in 169 dMMR CRC patients. It was obviously different from the prevalence of distant metastasis of dMMR CRC (3.5%).^10^ One of the important reasons might be that the metastasis of dMMR CRC is associated with specific genetic or downstream gene expression changes of the primary tumor.^10^ Both our findings and other studies have shown the distant metastatic rarity in dMMR CRC compared to approximately 50% distant metastasis in pMMR CRC. Furthermore, our meta‐analysis revealed that dMMR CRC patients experience a lower RFS rate compared to those with pMMR. While data limitations prevented us from specifically analyzing differences in DRFS, it's worth noting that RFS is a significant indicator of metastatic potential. This is underscored by the fact that in CRC, metastasis accounts for a substantial 78% of all recurrences.^23^ However, the underlying mechanisms of this low metastatic potential of dMMR CRC have yet to be elucidated.

Another strength of our study is the identification of signaling networks and hub genes potentially involved in inhibiting distant metastasis, enriched using WGCNA algorithms from gene microarray datasets. This identified 8 dMMR‐related modules, but only two modules (darkgreen and gray60) were strongly related to dMMR (cor = 0.58; *p* = 4e‐08 and cor = 0.31; *p* = 7.9e‐04 for dark green and gray 60, respectively) and distant metastasis (cor = 0.62; *p* = 2.3e‐09 and cor = 0.65; *p* = 5.1e‐15 for dark green and gray 60, respectively). The results indicate that darkgreen and gray60 modules were closely involved in the metastatic progression of CRC. The genes of the darkgreen and gray60 modules were correlated with metastasis‐related biological traits, including fibroblast migration,^24^ nuclear lamina^25^ and immunoactivation,^26^, ^27^ including antigen processing and presentation, and lymphocyte‐mediated immunity. At present, increasing evidence indicates that tumor dissemination is driven not only by single cells but also by cohesive cell groups, including cancer cells, fibroblasts and immune cells.^24^ Furthermore, our findings show that module genes linked to antigen processing, presentation, and lymphocyte‐mediated immunity have a strong association with dMMR but a negative correlation with metastasis. Several studies demonstrated that more tumor‐infiltrating lymphocytes were observed in dMMR tumors due to the generation of more neoantigens, thus activating immunoreaction. However, few studies have reported a greater immune reactivity of dMMR tumors, which may play a potential role in the inhibition of metastasis.^26^, ^27^ Therefore, whether antigen processing, presentation, and lymphocyte‐mediated immunity signaling are related to the process of distant metastasis requires further validation.

A total of 14 hub genes were identified in the darkgreen and gray60 modules based on the indexes of GS and MM. The expression of these 14 genes was validated in several independent datasets. Consensus expression of all the core genes was upregulated in dMMR patients and downregulated in distant metastasis patients. These core genes contained at least 8 (PPP2R2A, CXCL10, CXCL11, GBP1, IRF1, RARRES3, STAT1, and TAP1) previously identified regulators of tumor progression and distant metastasis. Interestingly, all 4 hub genes from the dark green module were located on chromosome 8p. Notably, 8p loss of heterozygosity (LOH) has been shown to be correlated with the higher metastatic potential of several types of cancers, and several 8p‐located genes have been implicated in tumor metastasis.^28^ However, of the 4 hub genes from the darkgreen module, only PPP2R2A was confirmed to enhance distant metastasis in thyroid cancer and pancreatic cancer.^29^, ^30^ In parallel, 7 genes (CXCL10, CXCL11, GBP1, IRF1, RARRES3, STAT1, TAP1) of the gray60 module play important dual roles in both immunoreaction and distant metastasis by activating either the epithelial‐mesenchymal transition pathway or T cell‐based immunosurveillance in several types of cancers.^31^, ^32^, ^33^ These immune‐related genes, RARRES3, CXCL11, IRF1, and STAT1, were also related to the metastatic behaviors of CRCs.^34^, ^35^, ^36^, ^37^

In addition, we found an interesting phenomenon in the present results. There were 3 transcription factors in metastasis‐related modules that could regulate 75% of the genes in their own module. Importantly, IRF1 and STAT1 were also hub genes of metastasis‐related modules. The two master regulatory transcription factors directly predict the regulation of the expression of a vast majority of the genes in the gene expression signature, suggesting a positive feedback loop. In particular, the transcription factor IRF1 can regulate not only more than 70% of genes in the metastasis‐related module but also all hub genes, including another transcription factor, STAT1. Thus, IRF1 might be a master transcription factor associated with a metastatic phenotype in CRC. Furthermore, the significance of distant metastasis of IRF1 was validated by univariable and multivariable analyses in the present study. IRF1 was significantly associated with RFS and DMFS in CRC, which has not been reported in previous CRC studies. While the role of IRF1 in distant metastasis was supported by mRNA expression data from several independent datasets and existing literature, further biological experiments are essential to confirm its actual role and underlying mechanism.

## 5 CONCLUSIONS

Our findings underscore that the distinctive loss patterns of MMR proteins could be influenced by geographical and ethnic considerations, with a notable prevalence of PSM2 loss, including its isolated loss. Furthermore, our study unveils a gene expression network linked to distant metastasis in CRC and emphasizes the pivotal regulatory role of IRF1. Further in‐depth biological studies are imperative to elucidate the precise roles and mechanisms of these identified markers, thereby advancing our therapeutic strategies for CRC patients.

## AUTHOR CONTRIBUTIONS

**Chuanwen Fan:** Conceptualization (equal); data curation (equal); formal analysis (equal); methodology (equal); writing – original draft (equal); writing – review and editing (equal). **Chao Fang:** Formal analysis (equal); methodology (equal); writing – original draft (equal). **Wei Wang:** Methodology (equal); writing – original draft (equal); writing – review and editing (equal). **Zhaoying Lv:** Formal analysis (equal); methodology (equal). **Xueli Zhang:** Formal analysis (equal). **Feiwu Long:** Investigation (equal). **Zongze Jiang:** Data curation (equal). **Yuan Li:** Formal analysis (equal); writing – review and editing (equal). **Hong Zhang:** Data curation (equal). **Zong‐Guang Zhou:** Conceptualization (equal); writing – review and editing (equal). **Cun Wang:** Conceptualization (equal); writing – original draft (equal); writing – review and editing (equal). **Xiao‐Feng Sun:** Conceptualization (equal); methodology (equal); writing – original draft (equal); writing – review and editing (equal).

## FUNDING INFORMATION

This work was supported by the Research Foundation of Outstanding Young Scholars of Sichuan University (Grant No. 2016SCU04B04, C Wang), Natural Science Foundation of Sichuan Province, China (Grant No. 2022NSFSC0764, CW Fan), the Fundamental Research Funds for the Central Universities (Grant No.2022SCU12025 and 2022SCU12020, CW Fan and FW Long), and Swedish Cancer Foundation and Swedish Research Council (XF Sun).

## CONFLICT OF INTEREST STATEMENT

The authors declare no conflict of interest.

## INSTITUTIONAL REVIEW BOARD STATEMENT

The study was conducted in accordance with the Declaration of Helsinki, and approved by the Ethics Committee of West China Hospital.

## CONSENT

Informed consent was obtained from all subjects involved in the study.

## Supporting information


Data S1.

Data S2.

Data S3.

Figure S1.
Figure S2.
Figure S3.
Figure S4.
Table S1.
Table S2.
Table S3.
Table S4.
Table S5.
Table S6.
Table S7.

## Data Availability

The TCGA data for CRC was obtained through https://portal.gdc.cancer.gov/. Additionally, we sourced the GSE39582, GSE39084, and GSE41258 datasets from the Gene Expression Omnibus (GEO) database, available at https://www.ncbi.nlm.nih.gov/geo/. Data from West China Hospital is also incorporated within this manuscript.
