# Supplementary material for: Mismatch repair protein deficiency and its implications on distant metastasis in colorectal cancer: A comprehensive analysis
Source: Cancer Med. 2024 Mar 28;13(7):e6994. doi: 10.1002/cam4.6994 (PMC10974709; doi:10.1002/cam4.6994)
Supplement: Supplementary file 4 — Figure S1. Figure S2. Figure S3. Figure S4. Table S1. Table S2. Table S3. Table S4. Table S5. Table S6. Table S7. [file CAM4-13-e6994-s001.docx]

**Supplementary**

**Mismatch Repair Protein Deficiency and Its Implications on Distant Metastasis in Colorectal Cancer: A Comprehensive Analysis**

**Authors:**

Chuanwen Fan ^1,2,3,4,6^, Wei Wang^2, 6^, Chao Fang ^1,3,6^, Zhaoying Lv ^1,6^, Xueli Zhang^5^, Feiwu Long^2^, Zongze Jiang^2^, Yuan Li ^1^, Hong Zhang ^5^, Zongguang Zhou ^1,3*^, Cun Wang ^3*^, and Xiao-Feng Sun^4*^

**Affiliations**

^1^ Institute of Digestive Surgery, Department of Gastrointestinal Surgery, West China Hospital, Sichuan Uni-versity, Chengdu, China.

^2^ Department of Gastrointestinal, Bariatric and Metabolic Surgery, Research Center for Nutrition, Metabolism & Food Safety, West China-PUMC C.C. Chen Institute of Health, West China School of Public Health and West China Fourth Hospital, Sichuan University, Chengdu, China.

^3^ Department of Gastrointestinal Surgery, State Key Laboratory of Biotherapy and Cancer Center, West China Hospital, Sichuan University, Chengdu, China.

^4^ Department of Oncology and Department of Clinical and Experimental Medicine, Linköping University, Linköping, Sweden.

^5^ Department of Medical Sciences, Örebro University, Örebro, Sweden.

^6^ These authors contributed equally

**Supplementary Figures**

**Figure S1**: Selection and evaluation of studies included in meta-analysis

**Figure S2**: Sensitivity analysis for the effect of each study on the pooled studies

**Figure S3:** WGCNA network and module detection

**Figure S4**: Preservation analyses of GSE41258 network modules in different datasets

**Supplementary Tables**

**Table S1:** Included studies in the meta-analysis

**Table S2**: Analysis of subgroup influences on study heterogeneity via meta-regression in CRC patients

**Table S3:** The modules identified by Weight gene coexpression network

**Table S4:** The absolute gene significance (GS) versus module membership (MM) of metastasis-related gene modules, and the chromosome localization of genes in darkgreen and gray60 module

**Table S5:** ClueGO-CluePedia functional analyses of darkgreen module

**Table S6:** ClueGO-CluePedia functional analyses of gray60 module

**Table S7:** The GS and MM of the hub gene in metastasis-related gene modules (darkgreen and gray60 module), and their chromosome localization

**Data S1.** Clinical data of the sample involved in gene expression analysis.

**Data S2.** The results of iRegulon analysis.

**Data S3.** The univariable Cox regression analysis of recurrence-free survival (RFS) and distal-metastasis-free survival (DMFS) in GSE39582, TCGA, and GSE39084 datasets.


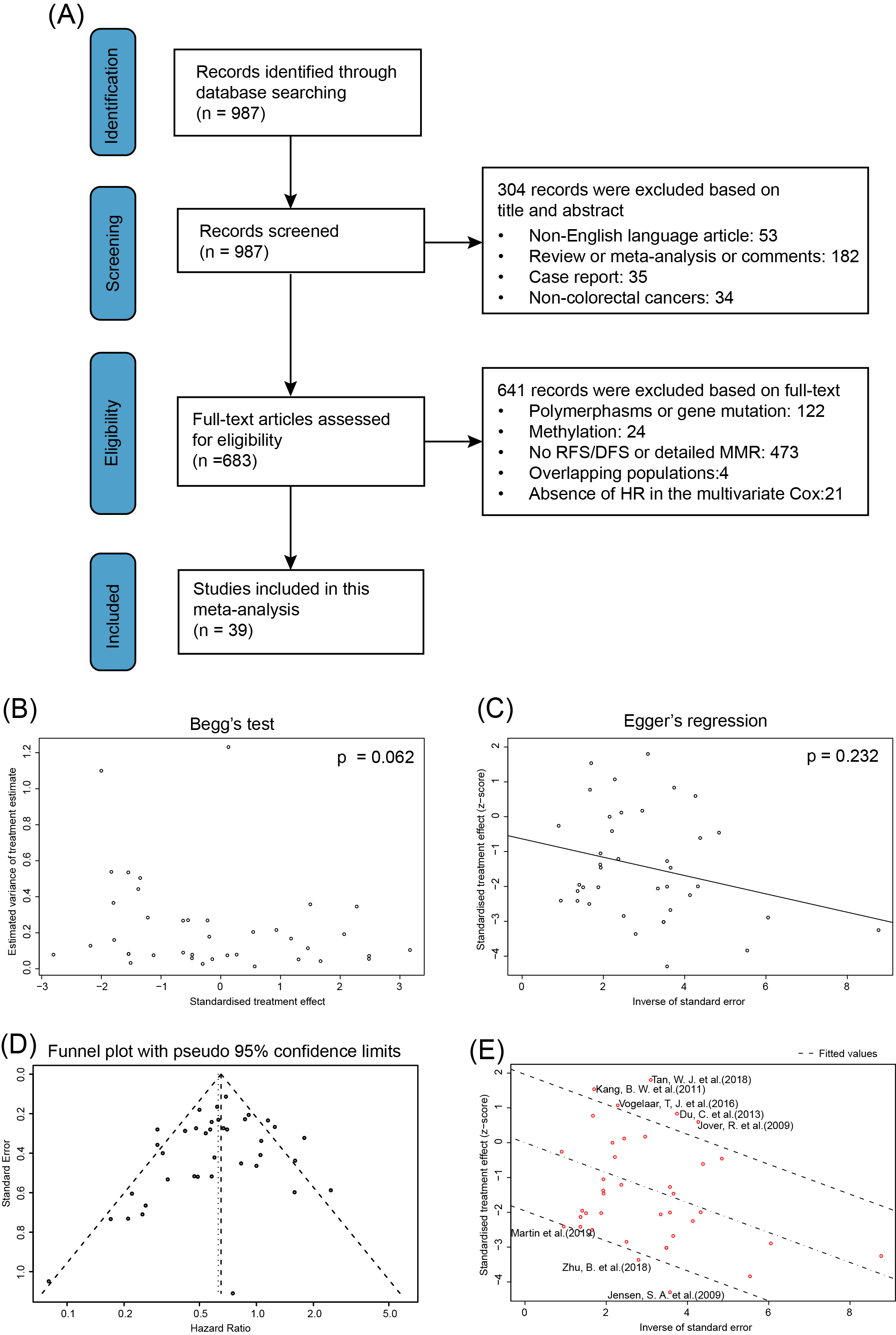


**Figure S1. Selection and evaluation of studies included in meta-analysis.** (A) Flowchart for selections of the studies included in the meta-analysis (B). The publication bias was detected by a Begg test. (C) The publication bias was detected by an Egger’s regression. (D) Funnel plot of recurrence free survival for estimation of publication bias. (E) Galbraith plot of association between MMR status and recurrence free survival in CRC patients.


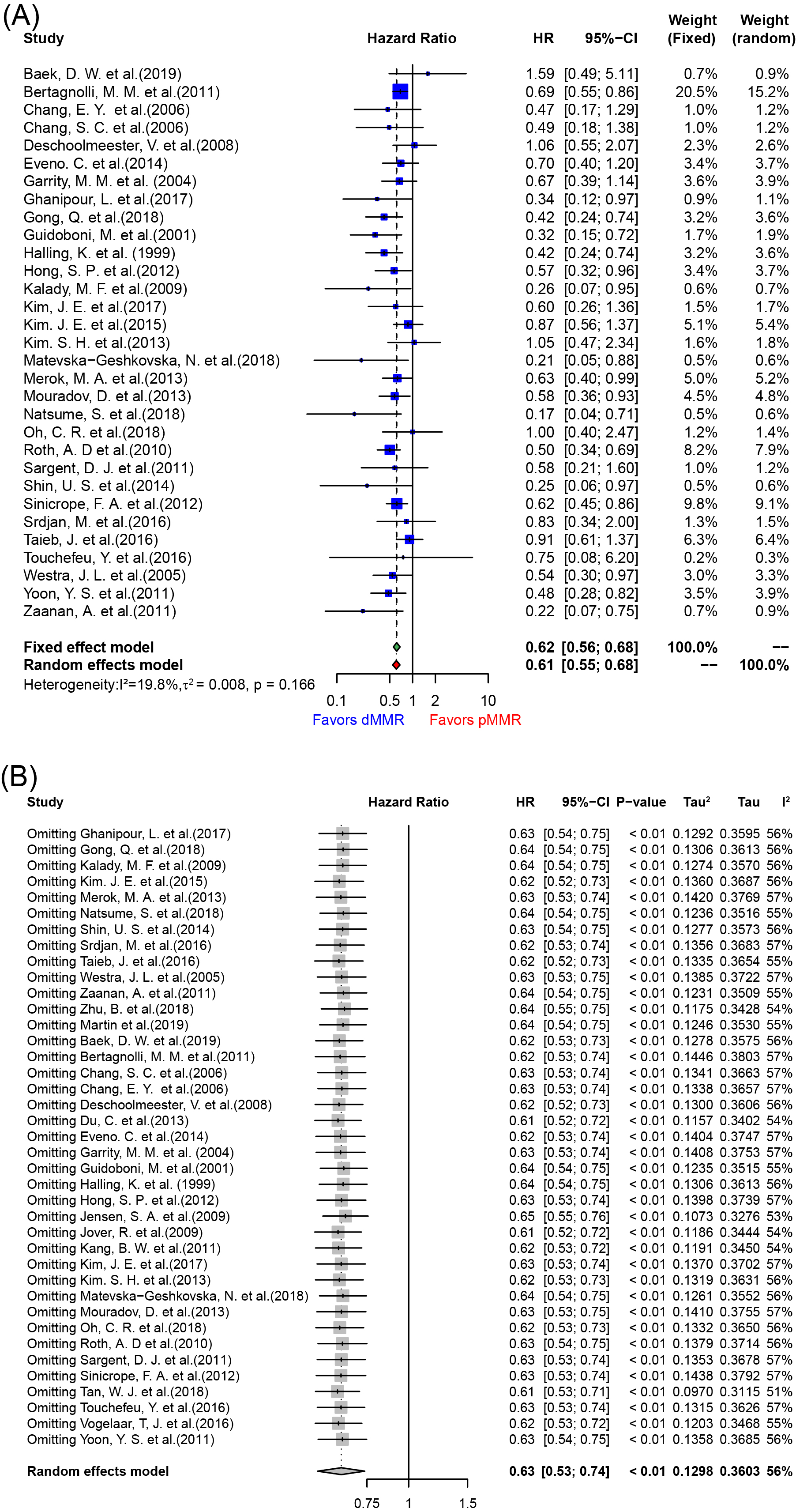


**Figure S2. Sensitivity analysis for the effect of each study on the pooled studies.** (A) Forest plots of HRs for recurrence-free survival in CRC studies associated with mismatch repair deficiency, after excluding eight studies with significant heterogeneity. (B) Forest plot depicting leave-one-out sensitivity analysis of recurrence-free survival in studies of CRC associated with mismatch repair deficiency.


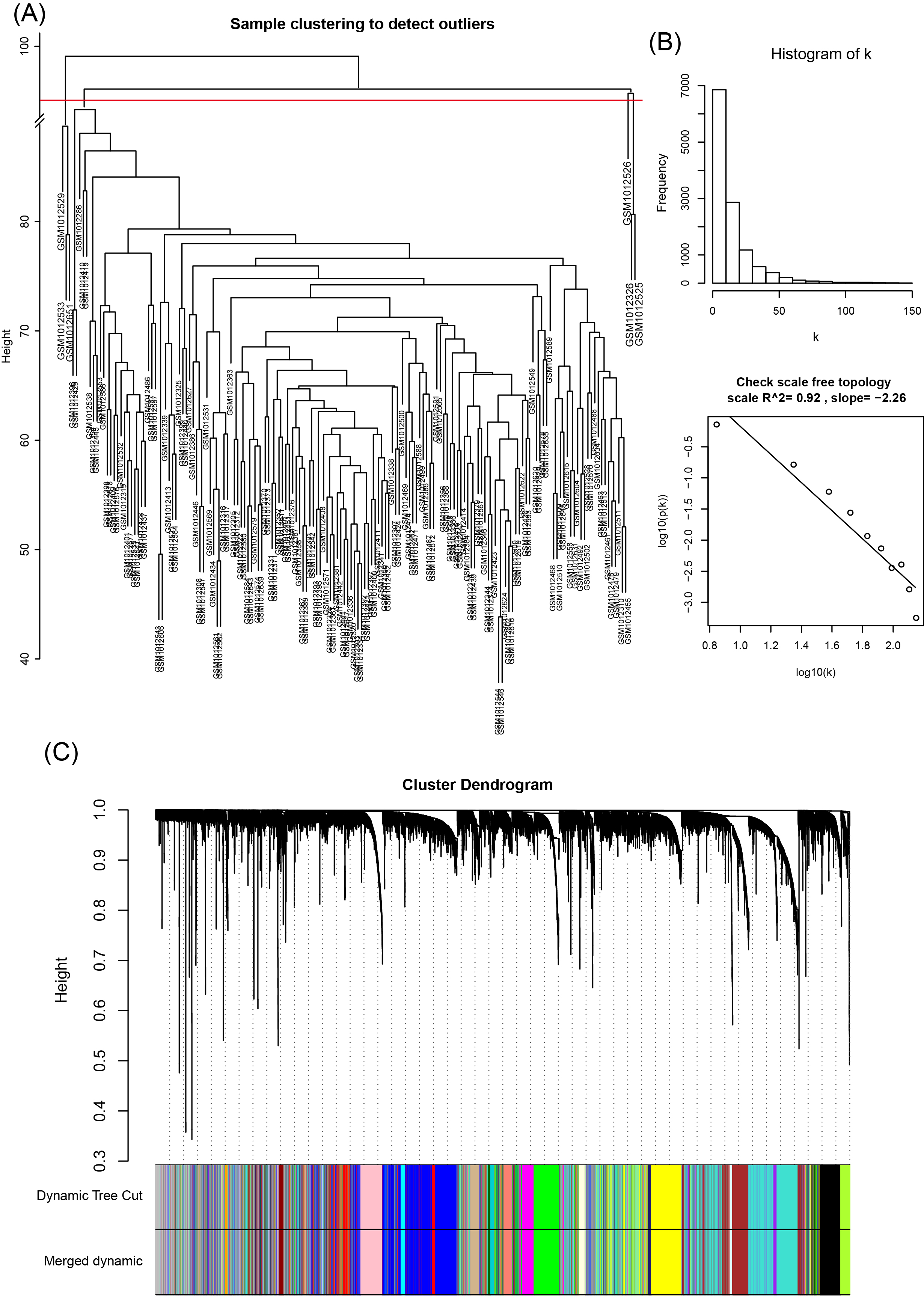


**Supplementary Figure S3.** **WGCNA network and module detection.** (A) Hierarchical average linkage clustering to detect outlier samples, red line repents 95**.** (B) Histogram of connectivity distribution and the scale-free topology when beta = 6. (C) Cluster dendrogram and module assignment for modules from WGCNA. Genes were clustered based on a dissimilarity measure (1-TOM). The branches correspond to modules of highly interconnected groups of genes. Colors in the horizontal bar represent the modules. 26 modules with 12,413 genes were detected with WGCNA.


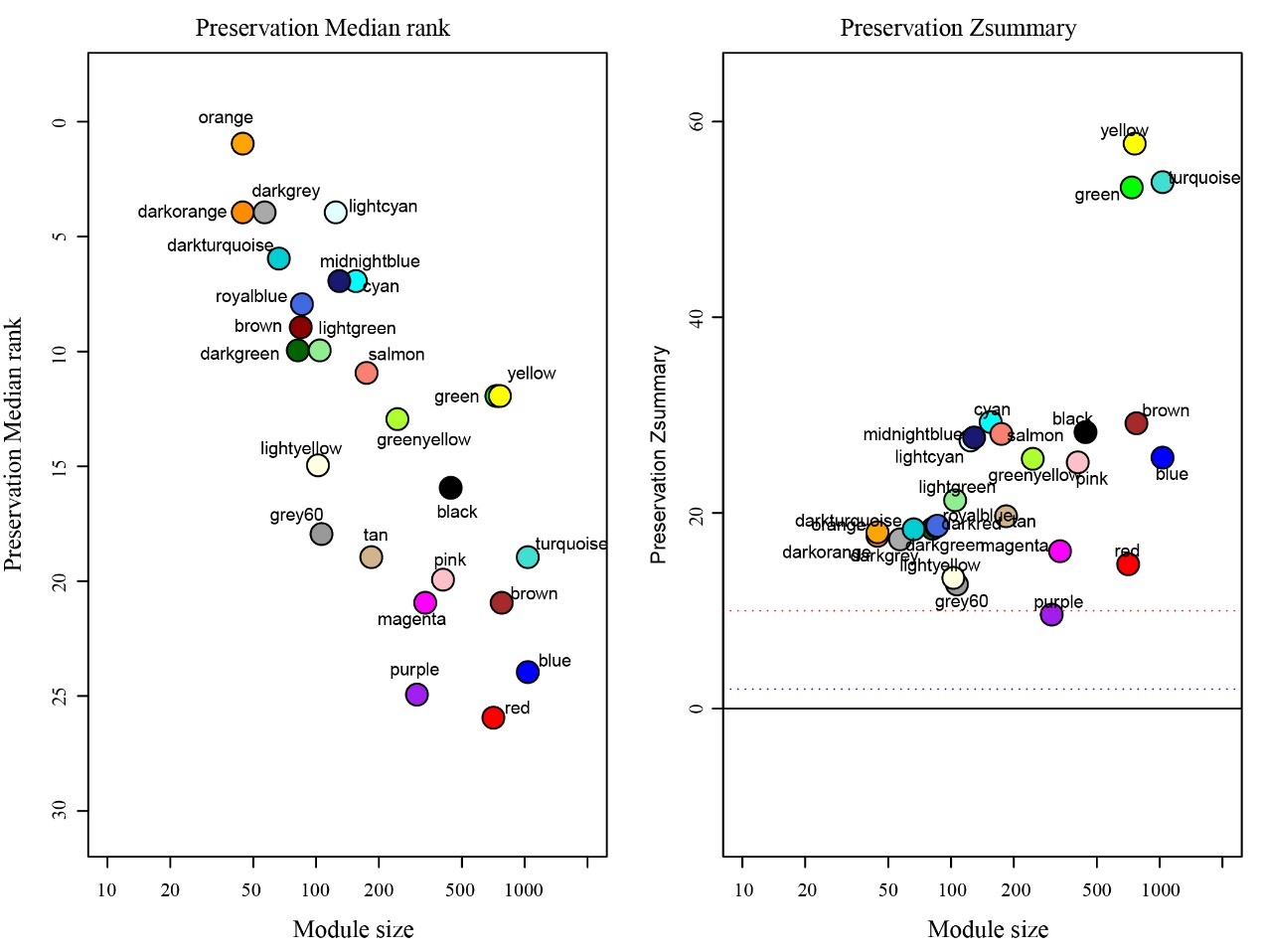


**Supplementary Figure S4.** **Preservation analyses of GSE41258 network modules in different datasets.** Each module was represented by its color code and name. The left figure showed the composite statistic preservation median rank. This measure tended to be independent of module size with high median ranks indicating low preservation. The right figure showed preservation Zsummary statistic. The dashed blue and green lines indicated the thresholds Z = 2 and Z = 10, respectively. Zsummary < 2 implied no evidence for module preservation, 2 < Zsummary < 10 implies weak to moderate evidence, and Zsummary > 10 implies strong evidence for module preservation. The metastasis-related modules (darkgreen and grey60) showed high preservation statistics summary than expected by random chance using bootstrapping validation procedures.

**Table S1. Included studies in the meta-analysis**

| **Study** | **HR (95% CI) in Multi** | **Compare group** | **Location** | **Country** | **Year** | **Stage** | **Total No.** | **No. of pMMR** | **No. of dMMR** | **Method of MSI determination** |
| --- | --- | --- | --- | --- | --- | --- | --- | --- | --- | --- |
| Baek, D | 1.59 (0.49; 5.11) | MSS | Colorectal cancer | Republic of Korea | 2019 | II | 237 | 161 | 76 | PCR/IHC |
| Bertagnolli, M. M | 0.69 (0.55; 0.86) | MSS | Colorectal cancer | USA | 2011 | II-III | 1845 | 1515 | 330 | PCR/IHC |
| Chang EY | 0.471(0.17; 1.29) | MSS | Colorectal cancer | Portland | 2006 | I-IV | 140 | 97 | 43 | PCR |
| Chang, S. C | 0.49 (0.18; 1.38) | MSS | Colorectal cancer | China | 2006 | I-IV | 213 | 194 | 19 | PCR |
| Deschoolmeester, V | 1.06 (0.55; 2.07) | MSS | Colon | Belgium | 2008 | I-III | 331 | 300 | 31 | IHC |
| Du, C., J | 1.25 (0.74; 2.11) | MSS | Rectum | China | 2013 | I-III | 316 | 291 | 25 | PCR |
| Eveno, C | 0.70 (0.40; 1.20) | MSS | Rectum | France | 2014 | I-III | 167 | 152 | 15 | IHC |
| Garrity, M. M | 0.67 (0.39; 1.14) | MSS | Colon | USA | 2004 | II-III | 364 | 310 | 54 | IHC |
| Ghanipour, L | 2.98 (1.03; 8.59) | MSI | Colorectal cancer | Sweden | 2017 | I-IV | 313 | 266 | 47 | PCR/IHC |
| Gong, Q | 2.37 (1.35; 4.17) | MSI | Colorectal cancer | China | 2018 | I-IV | 192 | 164 | 28 | IHC |
| Guidoboni, M | 0.32 (0.15; 0.72) | MSS | Colon | Italy | 2001 | II-III | 109 | 62 | 47 | PCR |
| Halling, K. C | 0.42 (0.24; 0.74) | MSS | Colorectal cancer | USA | 1999 | II-III | 411 | 335 | 76 | PCR |
| Hong, S. P | 0.57 (0.32; 0.96) | MSS | Colorectal cancer | Korea | 2012 | I-IV | 1125 | 1019 | 106 | PCR |
| Jensen, S. A. | 0.30 (0.20; 0.60) | MSS | Colorectal cancer | Denmark | 2009 | II-IV | 311 | 268 | 43 | IHC/PCR |
| Jover, R. | 1.15 (0.73; 1.83) | MSS | Colorectal cancer | Spain | 2009 | II-III | 505 | 445 | 60 | IHC/PCR |
| Kalady, M. F. | 3.86 (1.05 14.19) | MSI | Colorectal cancer | USA | 2009 | I-IV | 357 | 278 | 79 | PCR |
| Kang, B. W | 2.47 (0.78; 7.82) | MSS | Colorectal cancer | Korea | 2011 | II-III | 564 | 523 | 41 | PCR |
| Kim, J. E | 0.60 (0.26; 1.36) | MSS | Colon | Korea | 2017 | III | 598 | 548 | 50 | PCR |
| Kim, J. E. | 1.15 (0.73; 1.80) | MSI | Colon | Korea | 2015 | II | 860 | 734 | 126 | IHC |
| Kim, S. H | 1.05 (0.47; 2.34) | MSS | Colon | Korea | 2013 | III | 394 | 368 | 26 | PCR |
| Martin M. | 0.08 (0.01; 0.61) | MSS | Colorectal cancer | Norway | 2019 | I–III | 161 | 117 | 44 | PCR |
| Matevska-Geshkovska, N. | 0.21 (0.05; 0.88) | MSS | Colon | Mexico | 2018 | II-III | 96 | 79 | 17 | PCR |
| Merok, M. A | 1.60 (1.01; 2.52) | MSI | Colorectal cancer | Norway | 2013 | I-IV | 613 | 521 | 92 | PCR |
| Mouradov D | 0.58 (0.36; 0.93) | MSS | Colorectal cancer | UK | 2013 | II-III | 822 | 712 | 110 | PCR |
| Natsume, S. | 6.03 (1.41; 25.8) | MSI | Colorectal cancer | Japan | 2018 | I-IV | 575 | 541 | 34 | PCR |
| Oh, C. R | 1.00 (0.40; 2.47) | MSS | Colorectal cancer | Korea | 2018 | II-III | 1103 | 1079 | 24 | PCR |
| Roth, A. D | 0.54 (0.37; 0.81) | MSS | Colon | Switzerland | 2010 | II-III | 1254 | 1064 | 190 | PCR |
| Sargent, D. J | 0.58 (0.21; 1.60) | MSS | Colon | Canada/USA | 2011 | II | 229 | 153 | 76 | IHC |
| Shin, U. S. | 4.00 (1.03; 15.64) | MSI | Colorectal cancer | Korea | 2014 | II-III | 245 | 225 | 20 | PCR |
| Sinicrope, F. A. | 0.62 (0.45; 0.86) | MSS | Colon | USA | 2012 | II-III | 2693 | 2266 | 427 | PCR/IHC |
| Srdjan, M | 1.20(0.50; 2.90) | MSI | Colorectal cancer | Serbia | 2016 | II-III | 125 | 104 | 21 | PCR |
| Taieb, J | 1.10 (0.73; 1.64) | MSI | Colon | France | 2016 | III | 1791 | 1614 | 177 | PCR/IHC |
| Tan, W. J. | 1.79 (0.95; 3.37) | MSS | Colorectal cancer | Singapore | 2018 | I-IV | 654 | 591 | 63 | IHC |
| Touchefeu, Y | 0.75 (0.08; 6.20) | MSS | Colorectal cancer | France | 2016 | II | 187 | 137 | 50 | PCR |
| Vogelaar, F. J | 1.60 (0.70; 3.90) | MSS | Colon | Netherlands | 2016 | II | 166 | 143 | 23 | PCR |
| Westra JL | 1.80(0.97; 3.32) | MSI | Colon | Netherlands | 2005 | III | 273 | 229 | 44 | PCR |
| Yoon, Y. S. | 0.48 (0.28; 0.82) | MSS | Colorectal cancer | Korea | 2011 | I-IV | 1210 | 1092 | 118 | IHC/PCR |
| Zaanan, A. | 4.48 (1.34; 14.99) | MSI | Colon | France | 2011 | III | 303 | 269 | 34 | IHC/PCR |
| Zhu, B | 3.28 (1.65; 6.55) | MSI | Colorectal cancer | China | 2018 | I-IV | 337 | 274 | 63 | IHC |

Abbreviations: dMMR, deficient mismatch repair; IHC, immunohistochemistry; PCR, ​Polymerase Chain Reaction

**Table S2. Analysis of subgroup influences on study heterogeneity via meta-regression in CRC patients**

| **Subgroup variables** | **No. of records** | **Summary HR (95% CI)** | **Heterogeneity** | | **Meta-regression** | |
| --- | --- | --- | --- | --- | --- | --- |
|  |  |  | **I^2^ (%)** | **P value** | **R^2^** | **P value** |
| **Publication year** |  |  |  |  | 4.38 | 0.20 |
| 2010 or after | 28 | 0.67 (0.56; 0.86) | 53.80 | <0.01 |  |  |
| Before 2010 | 11 | 0.54 (0.40; 0.72) | 57.00 | <0.01 |  |  |
| **Tumor location** |  |  |  |  | 0.16 | 0.33 |
| colon | 14 | 0.56 (0.45; 0.74) | 44.20 | 0.04 |  |  |
| rectal | 2 | 0.66 (0.54; 0.81) ^*^ | 55.40 | 0.13 |  |  |
| colorectal | 23 | 0.94 (0.53; 1.66) | 60.50 | <0.01 |  |  |
| **Stage** |  |  |  |  | 29.08 | 0.06 |
| II | 5 | 0.96 (0.68; 1.36) ^*^ | 0.00 | 0.52 |  |  |
| III | 5 | 0.68 (0.46; 0.99) ^*^ | 43.50 | 0.13 |  |  |
| II/III | 13 | 0.63 (0.51; 0.78) | 51.10 | 0.02 |  |  |
| I-III | 4 | 0.88 (0.56; 1.36) | 61.90 | 0.05 |  |  |
| II-IV | 1 | 0.30 (0.17; 0.52) | — | — |  |  |
| I-IV | 11 | 0.50 (0.35; 0.70) | 55.40 | 0.01 |  |  |
| **Sample size** |  |  |  |  | 7.01 | 0.07 |
| <500 | 24 | 0.54 (0.42; 0.69) | 53.00 | <0.01 |  |  |
| ≥500 | 15 | 0.73 (0.60; 0.88) | 55.90 | <0.01 |  |  |
| **Method of MMR status measurement** |  |  |  |  | 0.00 | 0.70 |
| Genotyping | 22 | 0.60 (0.48; 0.75) | 46.70 | <0.01 |  |  |
| IHC | 8 | 0.71 (0.49; 1.03) | 64.50 | <0.01 |  |  |
| Genotyping/IHC | 9 | 0.61 (0.44; 0.86) | 67.50 | <0.01 |  |  |

Abbreviations: HR, hazard ratio; CI, confidence interval; MMR, mismatch repair; CRC, colorectal cancer

**Table S3. The number of genes in modules identified by WGCNA**

| Module numbers | Module colors | Gene number in corresponding module |
| --- | --- | --- |
| 1 | black | 579 |
| 2 | blue | 1684 |
| 3 | brown | 1217 |
| 4 | cyan | 145 |
| 5 | darkgreen | 76 |
| 6 | darkgrey | 47 |
| 7 | darkred | 90 |
| 8 | darkturquoise | 71 |
| 9 | green | 846 |
| 10 | greenyellow | 226 |
| 11 | grey | 866 |
| 12 | grey60 | 114 |
| 13 | lightcyan | 123 |
| 14 | lightgreen | 110 |
| 15 | lightyellow | 109 |
| 16 | magenta | 435 |
| 17 | midnightblue | 144 |
| 18 | orange | 46 |
| 19 | pink | 485 |
| 20 | purple | 314 |
| 21 | red | 613 |
| 22 | royalblue | 103 |
| 23 | salmon | 172 |
| 24 | tan | 175 |
| 25 | turquoise | 2549 |
| 26 | yellow | 1071 |

**Table S4. The location of genes in metastases-related modules**

| Gene symbol | Modules | Chromosome | Gene start (bp) | Gene end (bp) |
| --- | --- | --- | --- | --- |
| LPIN1 | darkgreen | 2 | 11677595 | 11827409 |
| GTF2E1 | darkgreen | 3 | 120742637 | 120783069 |
| HSPA4L | darkgreen | 4 | 127781821 | 127840733 |
| DNAJB6 | darkgreen | 7 | 157335381 | 157417439 |
| AGPAT5 | darkgreen | 8 | 6708642 | 6761503 |
| BIN3 | darkgreen | 8 | 22620418 | 22669148 |
| CA2 | darkgreen | 8 | 85463968 | 85481493 |
| CCDC25 | darkgreen | 8 | 27733316 | 27772653 |
| CHMP7 | darkgreen | 8 | 23243637 | 23262000 |
| CNOT7 | darkgreen | 8 | 17224966 | 17246878 |
| DCTN6 | darkgreen | 8 | 30156319 | 30183639 |
| DMTN | darkgreen | 8 | 22048995 | 22082527 |
| ELP3 | darkgreen | 8 | 28089673 | 28191156 |
| ENTPD4 | darkgreen | 8 | 23385783 | 23457695 |
| EPHX2 | darkgreen | 8 | 27490779 | 27545564 |
| EXTL3 | darkgreen | 8 | 28600469 | 28755599 |
| FAM160B2 | darkgreen | 8 | 22089150 | 22104911 |
| FDFT1 | darkgreen | 8 | 11795573 | 11839304 |
| FZD3 | darkgreen | 8 | 28494205 | 28574267 |
| GSR | darkgreen | 8 | 30678066 | 30727846 |
| GTF2E2 | darkgreen | 8 | 30578318 | 30658236 |
| INTS9 | darkgreen | 8 | 28767661 | 28890242 |
| KBTBD11 | darkgreen | 8 | 1973677 | 2006936 |
| KCTD9 | darkgreen | 8 | 25427847 | 25458476 |
| KIF13B | darkgreen | 8 | 29067278 | 29263124 |
| LEPROTL1 | darkgreen | 8 | 30095398 | 30177208 |
| MAK16 | darkgreen | 8 | 33485182 | 33501262 |
| MCPH1 | darkgreen | 8 | 6406596 | 6648508 |
| MFHAS1 | darkgreen | 8 | 8783354 | 8893630 |
| MSRA | darkgreen | 8 | 10054292 | 10428891 |
| MTUS1 | darkgreen | 8 | 17643795 | 17800917 |
| NAT1 | darkgreen | 8 | 18170477 | 18223689 |
| NUDT18 | darkgreen | 8 | 22106874 | 22109419 |
| PCM1 | darkgreen | 8 | 17922840 | 18029944 |
| POLR3D | darkgreen | 8 | 22245133 | 22254601 |
| PPP2R2A | darkgreen | 8 | 26291508 | 26372680 |
| PSD3 | darkgreen | 8 | 18527303 | 19084730 |
| PTK2B | darkgreen | 8 | 27311482 | 27459391 |
| R3HCC1 | darkgreen | 8 | 23270120 | 23296279 |
| REEP4 | darkgreen | 8 | 22138020 | 22141951 |
| SARAF | darkgreen | 8 | 30063012 | 30083208 |
| SH2D4A | darkgreen | 8 | 19313693 | 19396218 |
| SLC39A14 | darkgreen | 8 | 22367249 | 22434129 |
| SORBS3 | darkgreen | 8 | 22544986 | 22575788 |
| TNFRSF10B | darkgreen | 8 | 23020133 | 23069031 |
| TNKS | darkgreen | 8 | 9555912 | 9782346 |
| TTI2 | darkgreen | 8 | 33473386 | 33513601 |
| UBXN8 | darkgreen | 8 | 30732247 | 30767006 |
| WRN | darkgreen | 8 | 31033788 | 31175916 |
| XPO7 | darkgreen | 8 | 21919662 | 22006585 |
| ZNF395 | darkgreen | 8 | 28345590 | 28402701 |
| CASP7 | darkgreen | 10 | 113679162 | 113730907 |
| CUL5 | darkgreen | 11 | 108008898 | 108107761 |
| ADNP2 | darkgreen | 18 | 80109262 | 80147523 |
| CCDC68 | darkgreen | 18 | 54901509 | 54959461 |
| CNDP2 | darkgreen | 18 | 74495816 | 74523454 |
| FECH | darkgreen | 18 | 57544389 | 57586702 |
| GALNT1 | darkgreen | 18 | 35581117 | 35711834 |
| MALT1 | darkgreen | 18 | 58671386 | 58754477 |
| NARS | darkgreen | 18 | 57600656 | 57622213 |
| NDUFV2 | darkgreen | 18 | 9102630 | 9134345 |
| NOL4 | darkgreen | 18 | 33851100 | 34224952 |
| PIGN | darkgreen | 18 | 61905255 | 62187118 |
| RBFA | darkgreen | 18 | 80034389 | 80050651 |
| RNF138 | darkgreen | 18 | 32091874 | 32131561 |
| RPRD1A | darkgreen | 18 | 35984387 | 36067576 |
| SLC39A6 | darkgreen | 18 | 36108531 | 36129385 |
| SMAD2 | darkgreen | 18 | 47808957 | 47931146 |
| SOCS6 | darkgreen | 18 | 70289045 | 70330199 |
| TPGS2 | darkgreen | 18 | 36777647 | 36829216 |
| TRAPPC8 | darkgreen | 18 | 31829180 | 31953136 |
| TXNL1 | darkgreen | 18 | 56597209 | 56651600 |
| VPS4B | darkgreen | 18 | 63389190 | 63422483 |
| ZCCHC2 | darkgreen | 18 | 62523007 | 62587709 |
| ZNF24 | darkgreen | 18 | 35332227 | 35345482 |
| ATP5A1 | darkgreen | 18 | 46080248 | 46104233 |
| GBP1 | grey60 | 1 | 89052319 | 89065230 |
| RNF19B | grey60 | 1 | 32936445 | 32964685 |
| GNLY | grey60 | 2 | 85685175 | 85698854 |
| IFIH1 | grey60 | 2 | 162267074 | 162318703 |
| NMI | grey60 | 2 | 151270465 | 151290057 |
| SLC19A3 | grey60 | 2 | 227683763 | 227718028 |
| SP140L | grey60 | 2 | 230327184 | 230403732 |
| STAT1 | grey60 | 2 | 190964358 | 191020960 |
| VSNL1 | grey60 | 2 | 17539126 | 17657018 |
| ACOX2 | grey60 | 3 | 58505136 | 58537283 |
| LAMP3 | grey60 | 3 | 183122215 | 183163839 |
| MME | grey60 | 3 | 155024124 | 155183729 |
| PARP3 | grey60 | 3 | 51942345 | 51948867 |
| SCN5A | grey60 | 3 | 38548057 | 38649673 |
| SLC41A3 | grey60 | 3 | 126006355 | 126101561 |
| TMCC1 | grey60 | 3 | 129647792 | 129893606 |
| TNFSF10 | grey60 | 3 | 172505508 | 172523475 |
| AFP | grey60 | 4 | 73431138 | 73456174 |
| CXCL10 | grey60 | 4 | 76021118 | 76023497 |
| CXCL11 | grey60 | 4 | 76033682 | 76041415 |
| IL15 | grey60 | 4 | 141636583 | 141733987 |
| IRF2 | grey60 | 4 | 184387729 | 184474550 |
| LAP3 | grey60 | 4 | 17577192 | 17607972 |
| TLR3 | grey60 | 4 | 186069152 | 186088069 |
| IRF1 | grey60 | 5 | 132481609 | 132490777 |
| JADE2 | grey60 | 5 | 134524312 | 134583230 |
| BTN2A2 | grey60 | 6 | 26383096 | 26394874 |
| BTN3A1 | grey60 | 6 | 26402237 | 26415208 |
| BTN3A2 | grey60 | 6 | 26365159 | 26378320 |
| BTN3A3 | grey60 | 6 | 26440472 | 26453415 |
| CLIC5 | grey60 | 6 | 45880827 | 46080348 |
| ETV7 | grey60 | 6 | 36354091 | 36387800 |
| HCP5 | grey60 | 6 | 31400702 | 31477506 |
| HLA-A | grey60 | 6 | 29941260 | 29945884 |
| HLA-B | grey60 | 6 | 31269491 | 31357188 |
| HLA-C | grey60 | 6 | 31268749 | 31272130 |
| HLA-E | grey60 | 6 | 30489509 | 30494194 |
| HLA-F | grey60 | 6 | 29722775 | 29738528 |
| HLA-G | grey60 | 6 | 29826967 | 29831125 |
| HLA-J | grey60 | 6 | 30005971 | 30009956 |
| MICB | grey60 | 6 | 31494881 | 31511124 |
| POM121L2 | grey60 | 6 | 27285903 | 27312170 |
| PSMB8 | grey60 | 6 | 32840717 | 32844703 |
| PSMB9 | grey60 | 6 | 32844136 | 32859851 |
| TAP1 | grey60 | 6 | 32845209 | 32853978 |
| TAP2 | grey60 | 6 | 32821833 | 32838770 |
| TAPBP | grey60 | 6 | 33299694 | 33314387 |
| TRIM38 | grey60 | 6 | 25962802 | 25991231 |
| HCG26 | grey60 | 6 | 31471229 | 1472408 |
| GTF2IRD2B | grey60 | 7 | 75092396 | 75149817 |
| KBTBD2 | grey60 | 7 | 32868172 | 32894131 |
| ZNF273 | grey60 | 7 | 64870172 | 64930966 |
| IDO1 | grey60 | 8 | 39902275 | 39928790 |
| MTSS1 | grey60 | 8 | 124550790 | 124728429 |
| IFIT5 | grey60 | 10 | 89414568 | 89420997 |
| IL15RA | grey60 | 10 | 5943639 | 5978187 |
| SLC25A28 | grey60 | 10 | 99610522 | 99620609 |
| BIRC3 | grey60 | 11 | 102317450 | 102339403 |
| CASP1 | grey60 | 11 | 105025443 | 105035250 |
| IL18 | grey60 | 11 | 112143253 | 112164096 |
| NAV2 | grey60 | 11 | 19350724 | 20121601 |
| RARRES3 | grey60 | 11 | 63536808 | 63546462 |
| UBE2L6 | grey60 | 11 | 57551656 | 57568284 |
| PRB1 | grey60 | 12 | 11351823 | 11395566 |
| SLC11A2 | grey60 | 12 | 50979401 | 51028566 |
| SLCO1B3 | grey60 | 12 | 20810702 | 20916911 |
| TAPBPL | grey60 | 12 | 6451690 | 6466517 |
| GRK1 | grey60 | 13 | 113667219 | 113737736 |
| BTBD7 | grey60 | 14 | 93237550 | 93333092 |
| GZMB | grey60 | 14 | 24630954 | 24634267 |
| PSME1 | grey60 | 14 | 24136163 | 24138967 |
| RNASE3 | grey60 | 14 | 20891399 | 20892348 |
| SOS2 | grey60 | 14 | 50117130 | 50231578 |
| WARS | grey60 | 14 | 100333790 | 100376805 |
| B2M | grey60 | 15 | 44711487 | 44718877 |
| EHD4 | grey60 | 15 | 41895933 | 41972557 |
| ISG20 | grey60 | 15 | 88636153 | 88656483 |
| NEDD4 | grey60 | 15 | 55826922 | 55993746 |
| NOP10 | grey60 | 15 | 34341713 | 34343177 |
| PML | grey60 | 15 | 73994673 | 74047812 |
| CIITA | grey60 | 16 | 10866222 | 10943021 |
| CX3CL1 | grey60 | 16 | 57372477 | 57385044 |
| DEXI | grey60 | 16 | 10928891 | 10942468 |
| IL32 | grey60 | 16 | 3065297 | 3082192 |
| NUBP1 | grey60 | 16 | 10743786 | 10769351 |
| PSMB10 | grey60 | 16 | 67934506 | 67936864 |
| FDXR | grey60 | 17 | 74862497 | 74873031 |
| GSDMB | grey60 | 17 | 39904595 | 39919854 |
| LGALS9 | grey60 | 17 | 27629798 | 27649560 |
| SECTM1 | grey60 | 17 | 82321024 | 82334074 |
| PMAIP1 | grey60 | 18 | 59899948 | 59904306 |
| PQLC1 | grey60 | 18 | 79902420 | 79951657 |
| SMCHD1 | grey60 | 18 | 2655738 | 2805017 |
| TXNL4A | grey60 | 18 | 79970813 | 80033949 |
| C18orf8 | grey60 | 18 | 23503470 | 23531822 |
| OR7E19P | grey60 | 19 | 9265440 | 9268819 |
| PRKD2 | grey60 | 19 | 46674275 | 46717127 |
| SBNO2 | grey60 | 19 | 1107637 | 1174268 |
| ZNF253 | grey60 | 19 | 19865886 | 19894674 |
| ZNF254 | grey60 | 19 | 24033405 | 24129961 |
| ZNF675 | grey60 | 19 | 23525631 | 23687220 |
| ZNF85 | grey60 | 19 | 20923222 | 20950697 |
| ZNF223 | grey60 | 19 | 44051372 | 0.44069902 |
| ADA | grey60 | 20 | 44619522 | 44652233 |
| WFDC2 | grey60 | 20 | 45469753 | 45481532 |
| ZBP1 | grey60 | 20 | 57603846 | 57620576 |
| KCNJ6 | grey60 | 21 | 37607373 | 38121345 |
| TFF3 | grey60 | 21 | 42311667 | 42315651 |
| APOBEC3F | grey60 | 22 | 39040604 | 39055972 |
| APOL1 | grey60 | 22 | 36253010 | 36267530 |
| APOL2 | grey60 | 22 | 36226209 | 36239954 |
| APOL3 | grey60 | 22 | 36140330 | 36166177 |
| APOL6 | grey60 | 22 | 35648446 | 35668404 |
| SCO2 | grey60 | 22 | 50523568 | 50525606 |

**Table S5. The biological functions and pathway for darkgreen module**

| GO ID | GO Groups | Adjusted group *P* value | GO Term | Adjusted term *P* Value | Ontology Source | Associated Genes Found |
| --- | --- | --- | --- | --- | --- | --- |
| GO:0071498 | positive regulation of fibroblast migration | 3.34E-05 | cellular response to fluid shear stress | 3.99E-03 | GO_BiologicalProcess-EBI-UniProt-GOA_30.07.2018_00h00 | CA2, PTK2B |
| GO:1905476 | positive regulation of fibroblast migration | 3.34E-05 | negative regulation of protein localization to membrane | 7.67E-03 | GO_BiologicalProcess-EBI-UniProt-GOA_30.07.2018_00h00 | DMTN, FZD3 |
| GO:0010761 | positive regulation of fibroblast migration | 3.34E-05 | fibroblast migration | 8.08E-03 | GO_BiologicalProcess-EBI-UniProt-GOA_30.07.2018_00h00 | DMTN, PTK2B |
| GO:0010762 | positive regulation of fibroblast migration | 3.34E-05 | regulation of fibroblast migration | 6.23E-03 | GO_BiologicalProcess-EBI-UniProt-GOA_30.07.2018_00h00 | DMTN, PTK2B |
| GO:0060706 | positive regulation of fibroblast migration | 3.34E-05 | cell differentiation involved in embryonic placenta development | 6.40E-03 | GO_BiologicalProcess-EBI-UniProt-GOA_30.07.2018_00h00 | DNAJB6, PTK2B |
| GO:0010591 | positive regulation of fibroblast migration | 3.34E-05 | regulation of lamellipodium assembly | 7.33E-03 | GO_BiologicalProcess-EBI-UniProt-GOA_30.07.2018_00h00 | BIN3, DMTN |
| GO:0010763 | positive regulation of fibroblast migration | 3.34E-05 | positive regulation of fibroblast migration | 3.31E-03 | GO_BiologicalProcess-EBI-UniProt-GOA_30.07.2018_00h00 | DMTN, PTK2B |
| GO:1902001 | positive regulation of fibroblast migration | 3.34E-05 | fatty acid transmembrane transport | 3.91E-03 | GO_BiologicalProcess-EBI-UniProt-GOA_30.07.2018_00h00 | CA2, PTK2B |
| GO:0002335 | positive regulation of fibroblast migration | 3.34E-05 | mature B cell differentiation | 5.12E-03 | GO_BiologicalProcess-EBI-UniProt-GOA_30.07.2018_00h00 | MALT1, PTK2B |
| GO:0007250 | positive regulation of fibroblast migration | 3.34E-05 | activation of NF-kappaB-inducing kinase activity | 4.96E-03 | GO_BiologicalProcess-EBI-UniProt-GOA_30.07.2018_00h00 | MALT1, TNFRSF10B |
| GO:0071312 | nuclear lamina | 8.99E-05 | cellular response to alkaloid | 7.58E-03 | GO_BiologicalProcess-EBI-UniProt-GOA_30.07.2018_00h00 | CASP7, WRN |
| GO:0000729 | nuclear lamina | 8.99E-05 | DNA double-strand break processing | 5.47E-03 | GO_BiologicalProcess-EBI-UniProt-GOA_30.07.2018_00h00 | RNF138, WRN |
| GO:0005652 | nuclear lamina | 8.99E-05 | nuclear lamina | 2.72E-03 | GO_CellularComponent-EBI-UniProt-GOA_30.07.2018_00h00 | MTUS1, RNF138 |
| GO:0003954 | nuclear lamina | 8.99E-05 | NADH dehydrogenase activity | 8.16E-03 | GO_MolecularFunction-EBI-UniProt-GOA_30.07.2018_00h00 | NDUFV2, RNF138 |
| GO:0051537 | nuclear lamina | 8.99E-05 | 2 iron, 2 sulfur cluster binding | 4.96E-03 | GO_MolecularFunction-EBI-UniProt-GOA_30.07.2018_00h00 | FECH, NDUFV2 |
| GO:0016668 | oxidoreductase activity, acting on a sulfur group of donors, disulfide as acceptor | 4.09E-04 | oxidoreductase activity, acting on a sulfur group of donors, NAD(P) as acceptor | 4.07E-03 | GO_MolecularFunction-EBI-UniProt-GOA_30.07.2018_00h00 | GSR, TXNL1 |
| GO:0016671 | oxidoreductase activity, acting on a sulfur group of donors, disulfide as acceptor | 4.09E-04 | oxidoreductase activity, acting on a sulfur group of donors, disulfide as acceptor | 3.25E-03 | GO_MolecularFunction-EBI-UniProt-GOA_30.07.2018_00h00 | MSRA, TXNL1 |
| GO:0006998 | late endosome to vacuole transport | 4.83E-04 | nuclear envelope organization | 3.24E-03 | GO_BiologicalProcess-EBI-UniProt-GOA_30.07.2018_00h00 | CHMP7, LPIN1, REEP4 |
| GO:0045324 | late endosome to vacuole transport | 4.83E-04 | late endosome to vacuole transport | 2.25E-03 | GO_BiologicalProcess-EBI-UniProt-GOA_30.07.2018_00h00 | CHMP7, LEPROTL1 |
| GO:0071985 | late endosome to vacuole transport | 4.83E-04 | multivesicular body sorting pathway | 5.29E-03 | GO_BiologicalProcess-EBI-UniProt-GOA_30.07.2018_00h00 | LEPROTL1, VPS4B |
| GO:1904896 | late endosome to vacuole transport | 4.83E-04 | ESCRT complex disassembly | 2.25E-03 | GO_BiologicalProcess-EBI-UniProt-GOA_30.07.2018_00h00 | CHMP7, VPS4B |
| GO:0031468 | late endosome to vacuole transport | 4.83E-04 | nuclear envelope reassembly | 3.10E-03 | GO_BiologicalProcess-EBI-UniProt-GOA_30.07.2018_00h00 | CHMP7, REEP4 |
| GO:0032509 | late endosome to vacuole transport | 4.83E-04 | endosome transport via multivesicular body sorting pathway | 4.07E-03 | GO_BiologicalProcess-EBI-UniProt-GOA_30.07.2018_00h00 | LEPROTL1, VPS4B |
| GO:0036257 | late endosome to vacuole transport | 4.83E-04 | multivesicular body organization | 7.50E-03 | GO_BiologicalProcess-EBI-UniProt-GOA_30.07.2018_00h00 | CHMP7, VPS4B |
| GO:0007080 | late endosome to vacuole transport | 4.83E-04 | mitotic metaphase plate congression | 8.16E-03 | GO_BiologicalProcess-EBI-UniProt-GOA_30.07.2018_00h00 | CHMP7, VPS4B |
| GO:0036258 | late endosome to vacuole transport | 4.83E-04 | multivesicular body assembly | 7.41E-03 | GO_BiologicalProcess-EBI-UniProt-GOA_30.07.2018_00h00 | CHMP7, VPS4B |
| GO:0046755 | late endosome to vacuole transport | 4.83E-04 | viral budding | 5.38E-03 | GO_BiologicalProcess-EBI-UniProt-GOA_30.07.2018_00h00 | CHMP7, VPS4B |
| GO:1904903 | late endosome to vacuole transport | 4.83E-04 | ESCRT III complex disassembly | 2.25E-03 | GO_BiologicalProcess-EBI-UniProt-GOA_30.07.2018_00h00 | CHMP7, VPS4B |
| GO:0039702 | late endosome to vacuole transport | 4.83E-04 | viral budding via host ESCRT complex | 5.04E-03 | GO_BiologicalProcess-EBI-UniProt-GOA_30.07.2018_00h00 | CHMP7, VPS4B |
| GO:0097150 | microtubule anchoring at centrosome | 5.23E-04 | neuronal stem cell population maintenance | 5.12E-03 | GO_BiologicalProcess-EBI-UniProt-GOA_30.07.2018_00h00 | MCPH1, PCM1 |
| GO:0034453 | microtubule anchoring at centrosome | 5.23E-04 | microtubule anchoring | 5.04E-03 | GO_BiologicalProcess-EBI-UniProt-GOA_30.07.2018_00h00 | CCDC68, PCM1 |
| GO:0072393 | microtubule anchoring at centrosome | 5.23E-04 | microtubule anchoring at microtubule organizing center | 2.72E-03 | GO_BiologicalProcess-EBI-UniProt-GOA_30.07.2018_00h00 | CCDC68, PCM1 |
| GO:0034454 | microtubule anchoring at centrosome | 5.23E-04 | microtubule anchoring at centrosome | 2.25E-03 | GO_BiologicalProcess-EBI-UniProt-GOA_30.07.2018_00h00 | CCDC68, PCM1 |
| GO:1905508 | microtubule anchoring at centrosome | 5.23E-04 | protein localization to microtubule organizing center | 6.32E-03 | GO_BiologicalProcess-EBI-UniProt-GOA_30.07.2018_00h00 | MCPH1, PCM1 |
| GO:0000242 | microtubule anchoring at centrosome | 5.23E-04 | pericentriolar material | 4.07E-03 | GO_CellularComponent-EBI-UniProt-GOA_30.07.2018_00h00 | PCM1, TNKS |
| GO:0051721 | protein phosphatase 2A binding | 5.99E-04 | protein phosphatase 2A binding | 2.62E-03 | GO_MolecularFunction-EBI-UniProt-GOA_30.07.2018_00h00 | MFHAS1, PPP2R2A, PTK2B |
| GO:0016073 | snRNA metabolic process | 1.47E-03 | snRNA metabolic process | 2.12E-03 | GO_BiologicalProcess-EBI-UniProt-GOA_30.07.2018_00h00 | GTF2E1, GTF2E2, INTS9, RPRD1A |
| GO:0009301 | snRNA metabolic process | 1.47E-03 | snRNA transcription | 4.91E-03 | GO_BiologicalProcess-EBI-UniProt-GOA_30.07.2018_00h00 | GTF2E1, GTF2E2, INTS9, RPRD1A |
| GO:0042795 | snRNA metabolic process | 1.47E-03 | snRNA transcription by RNA polymerase II | 4.91E-03 | GO_BiologicalProcess-EBI-UniProt-GOA_30.07.2018_00h00 | GTF2E1, GTF2E2, INTS9, RPRD1A |
| GO:0005669 | snRNA metabolic process | 1.47E-03 | transcription factor TFIID complex | 6.91E-03 | GO_CellularComponent-EBI-UniProt-GOA_30.07.2018_00h00 | GTF2E1, GTF2E2 |
| KEGG:03022 | snRNA metabolic process | 1.47E-03 | Basal transcription factors | 7.50E-03 | KEGG_30.07.2018 | GTF2E1, GTF2E2 |
| GO:0048156 | tau protein binding | 2.18E-03 | tau protein binding | 4.07E-03 | GO_MolecularFunction-EBI-UniProt-GOA_30.07.2018_00h00 | PPP2R2A, SMAD2 |
| GO:0006829 | zinc ion import across plasma membrane | 1.60E-02 | zinc ion transport | 5.47E-03 | GO_BiologicalProcess-EBI-UniProt-GOA_30.07.2018_00h00 | SLC39A14, SLC39A6 |
| GO:0006882 | zinc ion import across plasma membrane | 1.60E-02 | cellular zinc ion homeostasis | 5.29E-03 | GO_BiologicalProcess-EBI-UniProt-GOA_30.07.2018_00h00 | SLC39A14, SLC39A6 |
| GO:0055069 | zinc ion import across plasma membrane | 1.60E-02 | zinc ion homeostasis | 5.47E-03 | GO_BiologicalProcess-EBI-UniProt-GOA_30.07.2018_00h00 | SLC39A14, SLC39A6 |
| GO:0071577 | zinc ion import across plasma membrane | 1.60E-02 | zinc ion transmembrane transport | 5.20E-03 | GO_BiologicalProcess-EBI-UniProt-GOA_30.07.2018_00h00 | SLC39A14, SLC39A6 |
| GO:0071578 | zinc ion import across plasma membrane | 1.60E-02 | zinc ion import across plasma membrane | 2.30E-03 | GO_BiologicalProcess-EBI-UniProt-GOA_30.07.2018_00h00 | SLC39A14, SLC39A6 |
| GO:0072509 | zinc ion import across plasma membrane | 1.60E-02 | divalent inorganic cation transmembrane transporter activity | 5.38E-03 | GO_MolecularFunction-EBI-UniProt-GOA_30.07.2018_00h00 | SLC39A14, SLC39A6 |
| GO:0046915 | zinc ion import across plasma membrane | 1.60E-02 | transition metal ion transmembrane transporter activity | 8.16E-03 | GO_MolecularFunction-EBI-UniProt-GOA_30.07.2018_00h00 | SLC39A14, SLC39A6 |
| GO:0005385 | zinc ion import across plasma membrane | 1.60E-02 | zinc ion transmembrane transporter activity | 4.88E-03 | GO_MolecularFunction-EBI-UniProt-GOA_30.07.2018_00h00 | SLC39A14, SLC39A6 |
| GO:0009164 | nucleoside diphosphate catabolic process | 2.37E-02 | nucleoside catabolic process | 7.50E-03 | GO_BiologicalProcess-EBI-UniProt-GOA_30.07.2018_00h00 | ENTPD4, NUDT18 |
| GO:0009134 | nucleoside diphosphate catabolic process | 2.37E-02 | nucleoside diphosphate catabolic process | 2.30E-03 | GO_BiologicalProcess-EBI-UniProt-GOA_30.07.2018_00h00 | ENTPD4, NUDT18 |
| GO:0009261 | nucleoside diphosphate catabolic process | 2.37E-02 | ribonucleotide catabolic process | 6.49E-03 | GO_BiologicalProcess-EBI-UniProt-GOA_30.07.2018_00h00 | ENTPD4, NUDT18 |
| GO:0042454 | nucleoside diphosphate catabolic process | 2.37E-02 | ribonucleoside catabolic process | 5.20E-03 | GO_BiologicalProcess-EBI-UniProt-GOA_30.07.2018_00h00 | ENTPD4, NUDT18 |
| GO:0009191 | nucleoside diphosphate catabolic process | 2.37E-02 | ribonucleoside diphosphate catabolic process | 2.59E-03 | GO_BiologicalProcess-EBI-UniProt-GOA_30.07.2018_00h00 | ENTPD4, NUDT18 |
| GO:0017110 | nucleoside diphosphate catabolic process | 2.37E-02 | nucleoside-diphosphatase activity | 3.20E-03 | GO_MolecularFunction-EBI-UniProt-GOA_30.07.2018_00h00 | ENTPD4, NUDT18 |

**Table S6. The biological functions and pathway for grey60 module**

| GO ID | GO Groups | Adjusted group *P* value | GO Term | Adjusted term *P* Value | Ontology Source | Associated Genes Found |
| --- | --- | --- | --- | --- | --- | --- |
| GO:0002250 | Antigen processing and presentation | 1.57E-13 | adaptive immune response | 5.95E-04 | GO_BiologicalProcess-EBI-UniProt-GOA_30.07.2018_00h00 | B2M, BTN3A2, BTN3A3, HLA-A, HLA-B, HLA-E, IL18 |
| GO:0002443 | Antigen processing and presentation | 1.57E-13 | leukocyte mediated immunity | 3.12E-05 | GO_BiologicalProcess-EBI-UniProt-GOA_30.07.2018_00h00 | B2M, BTN3A2, BTN3A3, GZMB, HLA-A, HLA-B, HLA-E, IL18, LGALS9 |
| GO:0019883 | Antigen processing and presentation | 1.57E-13 | antigen processing and presentation of endogenous antigen | 3.05E-10 | GO_BiologicalProcess-EBI-UniProt-GOA_30.07.2018_00h00 | B2M, HLA-A, HLA-B, HLA-C, HLA-E, TAP1, TAP2 |
| GO:0031342 | Antigen processing and presentation | 1.57E-13 | negative regulation of cell killing | 2.93E-04 | GO_BiologicalProcess-EBI-UniProt-GOA_30.07.2018_00h00 | HLA-A, HLA-B, HLA-E, LGALS9 |
| GO:0032609 | Antigen processing and presentation | 1.57E-13 | interferon-gamma production | 3.86E-05 | GO_BiologicalProcess-EBI-UniProt-GOA_30.07.2018_00h00 | BTN3A1, BTN3A2, BTN3A3, HLA-A, IL18, LGALS9, TLR3 |
| GO:0048002 | Antigen processing and presentation | 1.57E-13 | antigen processing and presentation of peptide antigen | 1.12E-09 | GO_BiologicalProcess-EBI-UniProt-GOA_30.07.2018_00h00 | B2M, HLA-A, HLA-B, HLA-C, HLA-E, TAP1, TAP2, TAPBPL |
| GO:0002697 | Antigen processing and presentation | 1.57E-13 | regulation of immune effector process | 6.25E-04 | GO_BiologicalProcess-EBI-UniProt-GOA_30.07.2018_00h00 | APOBEC3F, B2M, HLA-A, HLA-B, HLA-E, IL18, LGALS9, MICB, STAT1 |
| GO:0001819 | Antigen processing and presentation | 1.57E-13 | positive regulation of cytokine production | 1.66E-05 | GO_BiologicalProcess-EBI-UniProt-GOA_30.07.2018_00h00 | B2M, CASP1, HLA-A, HLA-E, HLA-G, IFIH1, IL15, IL18, IRF1, LGALS9, PRKD2, STAT1, TLR3 |
| GO:0001911 | Antigen processing and presentation | 1.57E-13 | negative regulation of leukocyte mediated cytotoxicity | 1.08E-04 | GO_BiologicalProcess-EBI-UniProt-GOA_30.07.2018_00h00 | HLA-A, HLA-B, HLA-E, LGALS9 |
| GO:0002449 | Antigen processing and presentation | 1.57E-13 | lymphocyte mediated immunity | 3.09E-06 | GO_BiologicalProcess-EBI-UniProt-GOA_30.07.2018_00h00 | B2M, BTN3A2, BTN3A3, GZMB, HLA-A, HLA-B, HLA-E, IL18, LGALS9 |
| GO:0002460 | Antigen processing and presentation | 1.57E-13 | adaptive immune response based on somatic recombination of immune receptors built from immunoglobulin superfamily domains | 2.06E-04 | GO_BiologicalProcess-EBI-UniProt-GOA_30.07.2018_00h00 | B2M, BTN3A2, BTN3A3, HLA-A, HLA-B, HLA-E, IL18 |
| GO:0002474 | Antigen processing and presentation | 1.57E-13 | antigen processing and presentation of peptide antigen via MHC class I | 3.05E-10 | GO_BiologicalProcess-EBI-UniProt-GOA_30.07.2018_00h00 | B2M, HLA-A, HLA-B, HLA-C, TAP1, TAP2, TAPBPL |
| GO:0002483 | Antigen processing and presentation | 1.57E-13 | antigen processing and presentation of endogenous peptide antigen | 1.63E-10 | GO_BiologicalProcess-EBI-UniProt-GOA_30.07.2018_00h00 | B2M, HLA-A, HLA-B, HLA-C, HLA-E, TAP1, TAP2 |
| GO:0002699 | Antigen processing and presentation | 1.57E-13 | positive regulation of immune effector process | 5.45E-03 | GO_BiologicalProcess-EBI-UniProt-GOA_30.07.2018_00h00 | B2M, HLA-A, HLA-B, HLA-E, IL18, LGALS9 |
| GO:0002228 | Antigen processing and presentation | 1.57E-13 | natural killer cell mediated immunity | 1.23E-03 | GO_BiologicalProcess-EBI-UniProt-GOA_30.07.2018_00h00 | GZMB, HLA-A, HLA-B, HLA-E, LGALS9 |
| GO:0002703 | Antigen processing and presentation | 1.57E-13 | regulation of leukocyte mediated immunity | 3.81E-03 | GO_BiologicalProcess-EBI-UniProt-GOA_30.07.2018_00h00 | B2M, HLA-A, HLA-B, HLA-E, IL18, LGALS9 |
| GO:0002819 | Antigen processing and presentation | 1.57E-13 | regulation of adaptive immune response | 9.05E-03 | GO_BiologicalProcess-EBI-UniProt-GOA_30.07.2018_00h00 | B2M, HLA-A, HLA-B, HLA-E, IL18 |
| GO:0042110 | Antigen processing and presentation | 1.57E-13 | T cell activation | 3.52E-03 | GO_BiologicalProcess-EBI-UniProt-GOA_30.07.2018_00h00 | ADA, BTN3A1, HLA-A, HLA-E, HLA-G, IL18, LGALS9, MICB |
| GO:0042267 | Antigen processing and presentation | 1.57E-13 | natural killer cell mediated cytotoxicity | 9.72E-04 | GO_BiologicalProcess-EBI-UniProt-GOA_30.07.2018_00h00 | GZMB, HLA-A, HLA-B, HLA-E, LGALS9 |
| GO:0002456 | Antigen processing and presentation | 1.57E-13 | T cell mediated immunity | 8.88E-06 | GO_BiologicalProcess-EBI-UniProt-GOA_30.07.2018_00h00 | B2M, BTN3A2, BTN3A3, HLA-A, HLA-B, HLA-E, IL18 |
| GO:0002704 | Antigen processing and presentation | 1.57E-13 | negative regulation of leukocyte mediated immunity | 1.83E-03 | GO_BiologicalProcess-EBI-UniProt-GOA_30.07.2018_00h00 | HLA-A, HLA-B, HLA-E, LGALS9 |
| GO:0002705 | Antigen processing and presentation | 1.57E-13 | positive regulation of leukocyte mediated immunity | 5.25E-03 | GO_BiologicalProcess-EBI-UniProt-GOA_30.07.2018_00h00 | B2M, HLA-A, HLA-B, HLA-E, IL18 |
| GO:0002821 | Antigen processing and presentation | 1.57E-13 | positive regulation of adaptive immune response | 1.38E-03 | GO_BiologicalProcess-EBI-UniProt-GOA_30.07.2018_00h00 | B2M, HLA-A, HLA-B, HLA-E, IL18 |
| GO:0019885 | Antigen processing and presentation | 1.57E-13 | antigen processing and presentation of endogenous peptide antigen via MHC class I | 1.37E-08 | GO_BiologicalProcess-EBI-UniProt-GOA_30.07.2018_00h00 | B2M, HLA-A, HLA-B, HLA-C, TAP1, TAP2 |
| GO:0002706 | Antigen processing and presentation | 1.57E-13 | regulation of lymphocyte mediated immunity | 9.24E-04 | GO_BiologicalProcess-EBI-UniProt-GOA_30.07.2018_00h00 | B2M, HLA-A, HLA-B, HLA-E, IL18, LGALS9 |
| GO:0002822 | Antigen processing and presentation | 1.57E-13 | regulation of adaptive immune response based on somatic recombination of immune receptors built from immunoglobulin superfamily domains | 5.70E-03 | GO_BiologicalProcess-EBI-UniProt-GOA_30.07.2018_00h00 | B2M, HLA-A, HLA-B, HLA-E, IL18 |
| GO:0042098 | Antigen processing and presentation | 1.57E-13 | T cell proliferation | 5.14E-03 | GO_BiologicalProcess-EBI-UniProt-GOA_30.07.2018_00h00 | BTN3A1, HLA-A, HLA-E, HLA-G, IL18, LGALS9 |
| GO:0002484 | Antigen processing and presentation | 1.57E-13 | antigen processing and presentation of endogenous peptide antigen via MHC class I via ER pathway | 3.68E-05 | GO_BiologicalProcess-EBI-UniProt-GOA_30.07.2018_00h00 | HLA-A, HLA-B, HLA-C |
| GO:0002707 | Antigen processing and presentation | 1.57E-13 | negative regulation of lymphocyte mediated immunity | 1.01E-03 | GO_BiologicalProcess-EBI-UniProt-GOA_30.07.2018_00h00 | HLA-A, HLA-B, HLA-E, LGALS9 |
| GO:0002708 | Antigen processing and presentation | 1.57E-13 | positive regulation of lymphocyte mediated immunity | 1.54E-03 | GO_BiologicalProcess-EBI-UniProt-GOA_30.07.2018_00h00 | B2M, HLA-A, HLA-B, HLA-E, IL18 |
| GO:0002715 | Antigen processing and presentation | 1.57E-13 | regulation of natural killer cell mediated immunity | 7.10E-03 | GO_BiologicalProcess-EBI-UniProt-GOA_30.07.2018_00h00 | HLA-A, HLA-B, HLA-E, LGALS9 |
| GO:0002824 | Antigen processing and presentation | 1.57E-13 | positive regulation of adaptive immune response based on somatic recombination of immune receptors built from immunoglobulin superfamily domains | 8.61E-04 | GO_BiologicalProcess-EBI-UniProt-GOA_30.07.2018_00h00 | B2M, HLA-A, HLA-B, HLA-E, IL18 |
| GO:0042269 | Antigen processing and presentation | 1.57E-13 | regulation of natural killer cell mediated cytotoxicity | 6.24E-03 | GO_BiologicalProcess-EBI-UniProt-GOA_30.07.2018_00h00 | HLA-A, HLA-B, HLA-E, LGALS9 |
| GO:0001916 | Antigen processing and presentation | 1.57E-13 | positive regulation of T cell mediated cytotoxicity | 3.01E-03 | GO_BiologicalProcess-EBI-UniProt-GOA_30.07.2018_00h00 | HLA-A, HLA-B, HLA-E |
| GO:0002709 | Antigen processing and presentation | 1.57E-13 | regulation of T cell mediated immunity | 6.68E-04 | GO_BiologicalProcess-EBI-UniProt-GOA_30.07.2018_00h00 | B2M, HLA-A, HLA-B, HLA-E, IL18 |
| GO:0002716 | Antigen processing and presentation | 1.57E-13 | negative regulation of natural killer cell mediated immunity | 1.08E-04 | GO_BiologicalProcess-EBI-UniProt-GOA_30.07.2018_00h00 | HLA-A, HLA-B, HLA-E, LGALS9 |
| GO:0045953 | Antigen processing and presentation | 1.57E-13 | negative regulation of natural killer cell mediated cytotoxicity | 1.08E-04 | GO_BiologicalProcess-EBI-UniProt-GOA_30.07.2018_00h00 | HLA-A, HLA-B, HLA-E, LGALS9 |
| GO:0046633 | Antigen processing and presentation | 1.57E-13 | alpha-beta T cell proliferation | 6.46E-04 | GO_BiologicalProcess-EBI-UniProt-GOA_30.07.2018_00h00 | HLA-A, HLA-E, IL18, LGALS9 |
| GO:0002486 | Antigen processing and presentation | 1.57E-13 | antigen processing and presentation of endogenous peptide antigen via MHC class I via ER pathway, TAP-independent | 3.68E-05 | GO_BiologicalProcess-EBI-UniProt-GOA_30.07.2018_00h00 | HLA-A, HLA-B, HLA-C |
| GO:0002711 | Antigen processing and presentation | 1.57E-13 | positive regulation of T cell mediated immunity | 6.61E-05 | GO_BiologicalProcess-EBI-UniProt-GOA_30.07.2018_00h00 | B2M, HLA-A, HLA-B, HLA-E, IL18 |
| GO:0042270 | Antigen processing and presentation | 1.57E-13 | protection from natural killer cell mediated cytotoxicity | 3.65E-04 | GO_BiologicalProcess-EBI-UniProt-GOA_30.07.2018_00h00 | HLA-A, HLA-B, HLA-E |
| GO:0046635 | Antigen processing and presentation | 1.57E-13 | positive regulation of alpha-beta T cell activation | 3.55E-03 | GO_BiologicalProcess-EBI-UniProt-GOA_30.07.2018_00h00 | HLA-A, HLA-E, IL18, LGALS9 |
| GO:2001198 | Antigen processing and presentation | 1.57E-13 | regulation of dendritic cell differentiation | 1.26E-03 | GO_BiologicalProcess-EBI-UniProt-GOA_30.07.2018_00h00 | HLA-B, HLA-G, LGALS9 |
| GO:0046640 | Antigen processing and presentation | 1.57E-13 | regulation of alpha-beta T cell proliferation | 6.46E-04 | GO_BiologicalProcess-EBI-UniProt-GOA_30.07.2018_00h00 | HLA-A, HLA-E, IL18, LGALS9 |
| GO:0046641 | Antigen processing and presentation | 1.57E-13 | positive regulation of alpha-beta T cell proliferation | 2.93E-04 | GO_BiologicalProcess-EBI-UniProt-GOA_30.07.2018_00h00 | HLA-A, HLA-E, IL18, LGALS9 |
| GO:0042611 | Antigen processing and presentation | 1.57E-13 | MHC protein complex | 2.16E-04 | GO_CellularComponent-EBI-UniProt-GOA_30.07.2018_00h00 | HLA-A, HLA-B, HLA-C, HLA-E |
| GO:0042612 | Antigen processing and presentation | 1.57E-13 | MHC class I protein complex | 2.26E-07 | GO_CellularComponent-EBI-UniProt-GOA_30.07.2018_00h00 | HLA-A, HLA-B, HLA-C, HLA-E |
| GO:0042824 | Antigen processing and presentation | 1.57E-13 | MHC class I peptide loading complex | 3.01E-03 | GO_CellularComponent-EBI-UniProt-GOA_30.07.2018_00h00 | TAP1, TAP2, TAPBP |
| GO:0042825 | Antigen processing and presentation | 1.57E-13 | TAP complex | 3.01E-03 | GO_CellularComponent-EBI-UniProt-GOA_30.07.2018_00h00 | TAP1, TAP2, TAPBP |
| GO:0046977 | Antigen processing and presentation | 1.57E-13 | TAP binding | 8.21E-11 | GO_MolecularFunction-EBI-UniProt-GOA_30.07.2018_00h00 | HLA-A, HLA-B, HLA-C, HLA-F, TAP1, TAP2, TAPBP |
| GO:0042605 | Antigen processing and presentation | 1.57E-13 | peptide antigen binding | 1.08E-04 | GO_MolecularFunction-EBI-UniProt-GOA_30.07.2018_00h00 | HLA-A, HLA-B, HLA-C, HLA-E |
| GO:0046978 | Antigen processing and presentation | 1.57E-13 | TAP1 binding | 7.26E-04 | GO_MolecularFunction-EBI-UniProt-GOA_30.07.2018_00h00 | HLA-F, TAP2, TAPBP |
| GO:0046979 | Antigen processing and presentation | 1.57E-13 | TAP2 binding | 7.26E-04 | GO_MolecularFunction-EBI-UniProt-GOA_30.07.2018_00h00 | HLA-F, TAP1, TAPBP |
| KEGG:04144 | Antigen processing and presentation | 1.57E-13 | Endocytosis | 4.94E-04 | KEGG_30.07.2018 | EHD4, GRK1, HLA-A, HLA-B, HLA-C, HLA-E, HLA-F, HLA-G, NEDD4, PML |
| KEGG:04145 | Antigen processing and presentation | 1.57E-13 | Phagosome | 8.07E-04 | KEGG_30.07.2018 | HLA-A, HLA-B, HLA-C, HLA-E, HLA-F, HLA-G, TAP1, TAP2 |
| KEGG:04612 | Antigen processing and presentation | 1.57E-13 | Antigen processing and presentation | 8.25E-12 | KEGG_30.07.2018 | B2M, CIITA, HLA-A, HLA-B, HLA-C, HLA-E, HLA-F, HLA-G, PSME1, TAP1, TAP2, TAPBP |
| KEGG:04650 | Antigen processing and presentation | 1.57E-13 | Natural killer cell mediated cytotoxicity | 2.12E-05 | KEGG_30.07.2018 | GZMB, HLA-A, HLA-B, HLA-C, HLA-E, HLA-G, MICB, SOS2, TNFSF10 |
| KEGG:04940 | Antigen processing and presentation | 1.57E-13 | Type I diabetes mellitus | 1.32E-06 | KEGG_30.07.2018 | GZMB, HLA-A, HLA-B, HLA-C, HLA-E, HLA-F, HLA-G |
| KEGG:05163 | Antigen processing and presentation | 1.57E-13 | Human cytomegalovirus infection | 2.72E-06 | KEGG_30.07.2018 | B2M, CX3CL1, HLA-A, HLA-B, HLA-C, HLA-E, HLA-F, HLA-G, SOS2, TAP1, TAP2, TAPBP |
| KEGG:05167 | Antigen processing and presentation | 1.57E-13 | Kaposi sarcoma-associated herpesvirus infection | 4.07E-04 | KEGG_30.07.2018 | HLA-A, HLA-B, HLA-C, HLA-E, HLA-F, HLA-G, MICB, STAT1, TLR3 |
| KEGG:05168 | Antigen processing and presentation | 1.57E-13 | Herpes simplex infection | 2.24E-08 | KEGG_30.07.2018 | HLA-A, HLA-B, HLA-C, HLA-E, HLA-F, HLA-G, IFIH1, IL15, PML, STAT1, TAP1, TAP2, TLR3 |
| KEGG:05320 | Antigen processing and presentation | 1.57E-13 | Autoimmune thyroid disease | 5.99E-06 | KEGG_30.07.2018 | GZMB, HLA-A, HLA-B, HLA-C, HLA-E, HLA-F, HLA-G |
| KEGG:05330 | Antigen processing and presentation | 1.57E-13 | Allograft rejection | 5.30E-07 | KEGG_30.07.2018 | GZMB, HLA-A, HLA-B, HLA-C, HLA-E, HLA-F, HLA-G |
| KEGG:05332 | Antigen processing and presentation | 1.57E-13 | Graft-versus-host disease | 9.30E-07 | KEGG_30.07.2018 | GZMB, HLA-A, HLA-B, HLA-C, HLA-E, HLA-F, HLA-G |
| KEGG:05416 | Antigen processing and presentation | 1.57E-13 | Viral myocarditis | 2.85E-04 | KEGG_30.07.2018 | HLA-A, HLA-B, HLA-C, HLA-E, HLA-F, HLA-G |
| GO:0002250 | lymphocyte mediated immunity | 3.60E-09 | adaptive immune response | 5.95E-04 | GO_BiologicalProcess-EBI-UniProt-GOA_30.07.2018_00h00 | B2M, BTN3A2, BTN3A3, HLA-A, HLA-B, HLA-E, IL18 |
| GO:0002443 | lymphocyte mediated immunity | 3.60E-09 | leukocyte mediated immunity | 3.12E-05 | GO_BiologicalProcess-EBI-UniProt-GOA_30.07.2018_00h00 | B2M, BTN3A2, BTN3A3, GZMB, HLA-A, HLA-B, HLA-E, IL18, LGALS9 |
| GO:0032609 | lymphocyte mediated immunity | 3.60E-09 | interferon-gamma production | 3.86E-05 | GO_BiologicalProcess-EBI-UniProt-GOA_30.07.2018_00h00 | BTN3A1, BTN3A2, BTN3A3, HLA-A, IL18, LGALS9, TLR3 |
| GO:0002449 | lymphocyte mediated immunity | 3.60E-09 | lymphocyte mediated immunity | 3.09E-06 | GO_BiologicalProcess-EBI-UniProt-GOA_30.07.2018_00h00 | B2M, BTN3A2, BTN3A3, GZMB, HLA-A, HLA-B, HLA-E, IL18, LGALS9 |
| GO:0002460 | lymphocyte mediated immunity | 3.60E-09 | adaptive immune response based on somatic recombination of immune receptors built from immunoglobulin superfamily domains | 2.06E-04 | GO_BiologicalProcess-EBI-UniProt-GOA_30.07.2018_00h00 | B2M, BTN3A2, BTN3A3, HLA-A, HLA-B, HLA-E, IL18 |
| GO:0042110 | lymphocyte mediated immunity | 3.60E-09 | T cell activation | 3.52E-03 | GO_BiologicalProcess-EBI-UniProt-GOA_30.07.2018_00h00 | ADA, BTN3A1, HLA-A, HLA-E, HLA-G, IL18, LGALS9, MICB |
| GO:0002456 | lymphocyte mediated immunity | 3.60E-09 | T cell mediated immunity | 8.88E-06 | GO_BiologicalProcess-EBI-UniProt-GOA_30.07.2018_00h00 | B2M, BTN3A2, BTN3A3, HLA-A, HLA-B, HLA-E, IL18 |
| GO:0042098 | lymphocyte mediated immunity | 3.60E-09 | T cell proliferation | 5.14E-03 | GO_BiologicalProcess-EBI-UniProt-GOA_30.07.2018_00h00 | BTN3A1, HLA-A, HLA-E, HLA-G, IL18, LGALS9 |
| GO:0050663 | lymphocyte mediated immunity | 3.60E-09 | cytokine secretion | 3.13E-03 | GO_BiologicalProcess-EBI-UniProt-GOA_30.07.2018_00h00 | BTN3A1, BTN3A2, BTN3A3, CASP1, CX3CL1, GBP1, IFIH1, LGALS9 |
| GO:0046633 | lymphocyte mediated immunity | 3.60E-09 | alpha-beta T cell proliferation | 6.46E-04 | GO_BiologicalProcess-EBI-UniProt-GOA_30.07.2018_00h00 | HLA-A, HLA-E, IL18, LGALS9 |
| GO:0072643 | lymphocyte mediated immunity | 3.60E-09 | interferon-gamma secretion | 1.25E-03 | GO_BiologicalProcess-EBI-UniProt-GOA_30.07.2018_00h00 | BTN3A1, BTN3A2, BTN3A3, LGALS9 |
| GO:0046635 | lymphocyte mediated immunity | 3.60E-09 | positive regulation of alpha-beta T cell activation | 3.55E-03 | GO_BiologicalProcess-EBI-UniProt-GOA_30.07.2018_00h00 | HLA-A, HLA-E, IL18, LGALS9 |
| GO:0046640 | lymphocyte mediated immunity | 3.60E-09 | regulation of alpha-beta T cell proliferation | 6.46E-04 | GO_BiologicalProcess-EBI-UniProt-GOA_30.07.2018_00h00 | HLA-A, HLA-E, IL18, LGALS9 |
| GO:0046641 | lymphocyte mediated immunity | 3.60E-09 | positive regulation of alpha-beta T cell proliferation | 2.93E-04 | GO_BiologicalProcess-EBI-UniProt-GOA_30.07.2018_00h00 | HLA-A, HLA-E, IL18, LGALS9 |
| GO:0045087 | innate immune response | 1.08E-08 | innate immune response | 2.00E-07 | GO_BiologicalProcess-EBI-UniProt-GOA_30.07.2018_00h00 | APOBEC3F, APOL1, BIRC3, CASP1, CIITA, GBP1, GZMB, HLA-A, HLA-B, HLA-E, IFIH1, LGALS9, PML, RNASE3, STAT1, TLR3 |
| GO:0009615 | defense response to other organism | 9.70E-08 | response to virus | 1.28E-04 | GO_BiologicalProcess-EBI-UniProt-GOA_30.07.2018_00h00 | APOBEC3F, IFIH1, IFIT5, IRF1, IRF2, ISG20, LGALS9, MICB, PMAIP1, STAT1 |
| GO:0098542 | defense response to other organism | 9.70E-08 | defense response to other organism | 3.33E-06 | GO_BiologicalProcess-EBI-UniProt-GOA_30.07.2018_00h00 | APOBEC3F, GNLY, HLA-A, HLA-E, IFIH1, IFIT5, IRF1, IRF2, ISG20, MICB, PMAIP1, RNASE3, STAT1 |
| GO:0050792 | defense response to other organism | 9.70E-08 | regulation of viral process | 5.71E-03 | GO_BiologicalProcess-EBI-UniProt-GOA_30.07.2018_00h00 | APOBEC3F, IFIT5, ISG20, LAMP3, LGALS9, PML, STAT1, TRIM38 |
| GO:0051607 | defense response to other organism | 9.70E-08 | defense response to virus | 2.58E-05 | GO_BiologicalProcess-EBI-UniProt-GOA_30.07.2018_00h00 | APOBEC3F, IFIH1, IFIT5, IRF1, IRF2, ISG20, MICB, PMAIP1, STAT1 |
| GO:1903900 | defense response to other organism | 9.70E-08 | regulation of viral life cycle | 8.55E-03 | GO_BiologicalProcess-EBI-UniProt-GOA_30.07.2018_00h00 | APOBEC3F, IFIT5, ISG20, LAMP3, LGALS9, PML, TRIM38 |
| KEGG:05164 | Influenza A | 1.09E-05 | Influenza A | 2.03E-04 | KEGG_30.07.2018 | CASP1, CIITA, CXCL10, IFIH1, IL18, PML, STAT1, TLR3, TNFSF10 |
| GO:0007249 | I-kappaB kinase/NF-kappaB signaling | 2.49E-05 | I-kappaB kinase/NF-kappaB signaling | 4.64E-04 | GO_BiologicalProcess-EBI-UniProt-GOA_30.07.2018_00h00 | BIRC3, CASP1, CX3CL1, IFIT5, STAT1, TLR3, TNFSF10, TRIM38, ZNF675 |
| GO:0045236 | CXCR chemokine receptor binding | 3.14E-04 | CXCR chemokine receptor binding | 5.85E-03 | GO_MolecularFunction-EBI-UniProt-GOA_30.07.2018_00h00 | CX3CL1, CXCL10, CXCL11 |
| GO:0034341 | response to interferon-gamma | 4.52E-04 | response to interferon-gamma | 8.41E-03 | GO_BiologicalProcess-EBI-UniProt-GOA_30.07.2018_00h00 | CASP1, CIITA, GBP1, LGALS9, STAT1 |

**Table S7. The hub genes and its GS and MM in metastases-related modules**

| Gene symbol | Modules | GS | P for GS | MM of darkgreen | P for MM of darkgreen | MM of grey60 | P value of grey60 MM | Chr | Gene start (bp) | Gene end (bp) |
| --- | --- | --- | --- | --- | --- | --- | --- | --- | --- | --- |
| GTF2E2 | darkgreen | 0.33 | 1.53E-05 | 0.81 | 1.46E-38 | 0.41 | 5.79E-08 | 8 | 30578318 | 30658236 |
| KCTD9 | darkgreen | 0.27 | 4.74E-04 | 0.81 | 9.39E-39 | 0.41 | 8.53E-08 | 8 | 25427847 | 25458476 |
| LEPROTL1 | darkgreen | 0.32 | 2.50E-05 | 0.81 | 6.55E-39 | 0.48 | 1.06E-10 | 8 | 30095398 | 30177208 |
| PPP2R2A | darkgreen | 0.31 | 4.60E-05 | 0.81 | 1.18E-38 | 0.40 | 1.24E-07 | 8 | 26291508 | 26372680 |
| APOL3 | grey60 | 0.20 | 9.19E-03 | 0.35 | 3.63E-06 | 0.82 | 4.82E-40 | 22 | 36140330 | 36166177 |
| CXCL10 | grey60 | 0.23 | 3.04E-03 | 0.37 | 1.27E-06 | 0.88 | 6.79E-53 | 4 | 76021118 | 76023497 |
| CXCL11 | grey60 | 0.30 | 1.07E-04 | 0.38 | 5.04E-07 | 0.84 | 5.83E-44 | 4 | 76033682 | 76041415 |
| GBP1 | grey60 | 0.21 | 6.50E-03 | 0.37 | 1.01E-06 | 0.87 | 6.81E-52 | 1 | 89052319 | 89065230 |
| IRF1 | grey60 | 0.26 | 8.06E-04 | 0.46 | 1.02E-09 | 0.87 | 3.92E-51 | 5 | 132481609 | 132490777 |
| PSMB9 | grey60 | 0.29 | 1.66E-04 | 0.31 | 4.52E-05 | 0.87 | 1.52E-50 | 6 | 32844136 | 32859851 |
| RARRES3 | grey60 | 0.22 | 5.32E-03 | 0.38 | 6.20E-07 | 0.86 | 4.22E-49 | 11 | 63536808 | 63546462 |
| STAT1 | grey60 | 0.20 | 9.34E-03 | 0.36 | 2.59E-06 | 0.86 | 5.22E-49 | 2 | 190964358 | 191020960 |
| TAP1 | grey60 | 0.27 | 4.66E-04 | 0.38 | 7.46E-07 | 0.91 | 1.52E-62 | 6 | 32845209 | 32853978 |
| UBE2L6 | grey60 | 0.24 | 2.15E-03 | 0.33 | 2.28E-05 | 0.88 | 2.27E-52 | 11 | 57551656 | 57568284 |

GS: gene significance, MM: module membership

**Data S1.** Clinical data of the sample involved in gene expression analysis.

Data S1 was listed in a separate file.

**Data S2.** The results of iRegulon analysis.

Data S2 was listed in a separate file.

**Data S3.** The univariable Cox regression analysis of recurrence-free survival (RFS) and distal-metastasis-free survival (DMFS) in GSE39582, TCGA, and GSE39084 datasets.

Data S3 was listed in a separate file.
